# Rarely reported cases of hepatotoxicity associated with turmeric- and curcuminoid-containing dietary supplements: a comprehensive review by USP

**DOI:** 10.1080/13880209.2026.2693375

**Published:** 2026-06-27

**Authors:** Nadeem Akhtar, Joanne Barnes, Paula Gardiner, Bill J. Gurley, Richard Ko, Igor Koturbash, Deval Patel, Richard B. van Breemen, Amy L. Roe

**Affiliations:** ^a^ United States Pharmacopeia-India (P) Ltd, Shamirpet, India; ^b^ Dietary Supplement Admission Evaluation & Labeling Expert Committee, United States Pharmacopeia, Rockville, MD, USA

**Keywords:** Curcumin, dietary supplement, hepatotoxicity, turmeric

## Abstract

**Background:**

The United States Pharmacopeia (USP) Dietary Supplement Admission Evaluation and Labeling Expert Committee (DSAEL EC) routinely monitors safety‑related literature for dietary ingredients covered by USP monographs. Following multiple reports of hepatotoxicity associated with turmeric- or curcuminoid-containing products, the DSAEL EC conducted a comprehensive evaluation.

**Objective:**

To assess the safety of turmeric and curcuminoids and determine whether cautionary labeling should be added to the USP monographs for these ingredients.

**Methods:**

Following USP Guidelines, the DSAEL EC reviewed safety and toxicological information.

**Results and Discussion:**

Hepatotoxicity has been observed with high-dose turmeric rhizome powder or extract in a few rodent toxicology studies. Numerous clinical trials involving turmeric- or curcuminoid-containing supplements have not reported organ toxicity or serious adverse events. Published case reports of clinically apparent liver injury often involve concomitant medications or supplements. Reported hepatotoxicity typically occurs after 1 to 4 months of use and generally resolves upon discontinuation; cases of acute liver failure have been reported albeit rarely. Proposed mechanisms include idiosyncratic, immune-mediated injury, and emerging evidence suggests possible genetic susceptibility.

**Conclusions:**

The USP DSAEL EC recommends adding the following cautionary statement to its turmeric and curcuminoids monographs: *Liver problems have been reported very rarely in people taking supplements containing turmeric and/or curcuminoids. Consult your health-care practitioner before using this product if you have a history of liver problems. Stop using this product if you develop symptoms such as abdominal pain, dark urine, or jaundice (yellowing of the skin or eyes), and seek medical advice.*

## Introduction

Turmeric (*Curcuma longa* L., Zingiberaceae) is a rhizome-derived botanical widely used as a culinary spice, traditional remedy, and dietary supplement. The rhizome contains nonvolatile curcuminoids (predominantly curcumin, with demethoxy- and bisdemethoxycurcumin) and volatile oils, and curcuminoids have been reported to comprise approximately 2–9% of turmeric depending on origin and cultivation conditions. Because orally consumed turmeric has been reported to have very low bioavailability (<1%), concentrated and ‘bioavailability-enhanced’ curcumin preparations, including products combined with piperine and lipid, nano-emulsion, or liposomal systems, are now widely available. In parallel, increasing pharmacovigilance and case-series evidence link turmeric- or curcuminoid-containing supplements to (predominantly) hepatocellular liver injury that typically improves after discontinuation, but which can also result in serious outcomes (including hospitalization and rare acute liver failure). This issue warrants a focused evaluation of the safety profile of turmeric and curcuminoids across contemporary formulations and exposure contexts (Iweala et al. 2023; Philips and Theruvath 2024).

An admission evaluation for turmeric (C. longa L., Zingiberaceae) and curcuminoids was conducted in October 2025 by the United States Pharmacopeia (USP) Dietary Supplement Admission Evaluation and Labeling Expert Committee (DSAEL EC), in accordance with the USP Guideline for the Admission of Dietary Supplement Ingredients to the USP–National Formulary (USP–NF) Monograph Development Process (USP 2009). Monographs associated with turmeric and curcuminoids currently exist in the USP–NF (USP-NFa-d). Recently, due to multiple case reports of hepatotoxicity related to turmeric and/or curcuminoids, along with regulatory reviews, expert opinions, and implementation of new labeling requirements from various drug regulatory authorities, the DSAEL EC conducted a comprehensive evaluation. The purpose was to advise the USP Botanical Dietary Supplements and Herbal Medicines Expert Committee (BDSHM EC) on whether any safety concerns exist, or if a cautionary label statement is warranted, for turmeric and/or curcuminoids monographs. The evaluation conducted by the DSAEL EC does not include a review of efficacy studies in clinical or animal models, except where such studies contain relevant safety information. The primary objective of the evaluation was to identify signals of serious risk to human health, and whether any risks warrant cautionary labeling for these dietary supplement ingredients.

Although New Dietary Ingredient Notifications (NDINs) and a Generally Recognized as Safe (GRAS) notice have been submitted to the U.S. Food and Drug Administration (FDA) for turmeric and/or curcuminoids (US FDA NDINs from 2005, 2006, 2007; US FDA GRAS notices issued in 2013, 2017, 2019), a comprehensive safety review was still deemed necessary. This is because of the wide variability in marketed multi‑ingredient products and formulations, and the availability of high-dose supplements in the market, which may not be adequately covered by GRAS or NDIN submissions.

The current *USP–NF* monographs and definitions related to turmeric and curcuminoids are listed in Table 1. Turmeric (*C. longa*) is also known as *C. domestica* Val. [synonym], and commonly known as curcuma, curcum, haridra, and Indian Saffron, among hundreds of other names [MPNS] Medicinal Plant Names Services, Royal Botanic Gardens, Kew). The *Curcuma* genus comprises approximately 70 rhizomatous species distributed across tropical and subtropical regions. The dried rhizomes of turmeric can be reduced to a powdered form. Turmeric rhizomes are also pulverized and extracted with various suitable solvents to form powdered turmeric extracts. Curcuminoids are a partially purified natural complex of diaryl heptanoid derivatives isolated from turmeric, and include curcumin, demethoxycurcumin, and bisdemethoxycurcumin.

**Table 1.** USP-NF Turmeric and curcuminoids monographs and definitions.

**Turmeric** is the dried rhizome of *C. longa* L., also known as *C. domestica* Val. (Fam. Zingiberaceae) containing not less than (NLT) 3.0% curcuminoids (as the sum of curcumin, desmethoxycurcumin, and bisdesmethoxycurcumin) on an anhydrous basis
**Powdered Turmeric** is turmeric reduced to a fine powder, containing NLT 3.0% curcuminoids (same specification as above for turmeric)
**Powdered Turmeric Extract** is prepared from pulverized rhizome of *C. longa* L., using acetone, methanol, or other suitable solvents. It contains NLT 20% curcuminoids (on dried basis). It may contain other added substances
**Curcuminoids** is a partially purified natural complex of diaryl heptanoid derivatives isolated from turmeric, *C. longa* L. The monograph specifies NLT 95.0% of curcuminoids, calculated on the dried basis, as the sum of the following, NLT 70% and not more than (NMT) 80% of curcumin, NLT 15.0% and NMT 25% of desmethoxycurcumin, and NLT 2.5% and NMT 6.5% of bisdesmethoxycurcumin
NB. Desmethoxycurcumin and bisdesmethoxycurcumin are also referred to as demethoxycurcumin and bisdemethoxycurcumin, respectively.

Turmeric, which has been used for over 4000 years, is widely utilized as a spice, preservative, and traditional medicine for gastrointestinal, hepatic, and skin disorders (Prasad and Aggarwal 2011). To date, no safety concern has been identified for turmeric when used as a spice or consumed in foods as part of a typical diet. The average dietary intake for turmeric in India is 2.0–2.5 g/day, providing 60–100 mg curcumin. Supplements typically use extracts standardized to ≥90% curcuminoids, and common dosages may be up to 3,000 mg/day (Table 2) (BfR. German Federal Institute for Risk Assessment (BfR) 2021; [DSLD] Dietary Supplement Label Database 2026; Supplement OWL (Online Wellness Library)) 2026). Numerous dietary supplement products are available in the marketplace, including those that claim enhanced bioavailability of curcuminoids. Given the nature of the health claims associated with turmeric- and curcuminoid-containing products, such as purported anti-inflammatory, anti-aging, and joint-health benefits, these products may be used for extended periods, potentially resulting in long durations of exposure.

**Table 2.** Typical intake levels based upon in-market search of dietary supplement products and traditional uses and exposure from dietary sources.

Source	Product type	Turmeric content per serving	Curcuminoid content
DSLD	1000+ products	6.25 mg–9,000 mg (400 mg–1500 mg most common)	3%–97% (most common 95%)
Supplement OWL	300+ products	25 mg–3,000 mg (100 mg–1000 mg most common)	20%–95% (most common 95%)
Amazon.com	500+ products	300 mg −3,000 mg (200 mg −1,000 mg most common)	Most common 95%
**Category**	**Source**	**Details**	
Traditional Dosage (Turmeric rhizome)	WHO Monograph	Crude: 3 g/day–9 g/day; Powdered: 1.5 g/day–3 g/day	
	Ayurvedic Pharmacopeia	Powder: 1 g/day–3 g/day	
	German Commission E	1.5 g/day–3 g/day	
	EMA Monograph	0.5 g–1 g, taken 2–3 times daily	
Dietary Exposure	Average Indian Intake	2g/day–2.5 g/day turmeric ≈ 60 mg–100 mg curcumin	
	EFSA (2010 and 2014)	Adults: 0.8 mg/kg bw/day (within ADI); Children: up to 3.8 mg/kg bw/day (some exceed ADI)	
	UK COT	Online sources suggest 500 mg/day–1,000 mg/day curcuminoids (may exceed ADI)	

Turmeric- and curcuminoid-containing products are available in multiple pharmaceutical forms, most commonly oral solids (capsules, tablets, pills, and soft gels), as well as tinctures and oral sprays, and topical or mucosal preparations (ointments, gels, creams, mouthwashes, and cosmetics). In dietary supplements marketed in the USA, products are often capsule-based and typically contain turmeric-derived curcuminoid extracts, sometimes combined with turmeric root and claimed bioavailability enhancers (e.g., piperine or proprietary complexes); less commonly, clinical use has included rectal suppositories and intravenous curcumin solutions. Formulations include conventional turmeric root preparations or curcuminoid extracts, as well as claimed ‘enhanced bioavailability’ systems, such as adjuvant-based products (e.g., piperine and/or turmeric oils or essential oils), solubility-focused delivery systems (e.g., micelles, emulsions, nanoparticles, liposomes, phospholipid or phytosomal complexes, and other lipid-based formulations), and natural-matrix approaches intended to improve delivery of free curcuminoids (e.g., galactomannan complexes or solid lipid-type systems) (Hegde et al. 2023; Skiba et al. 2018).

Considering this diversity, rigorous quality control is essential because commercial turmeric and curcuminoid products show substantial variability, including finished products and particularly extracts (Chatzinasiou et al. 2019). Accordingly, quality frameworks emphasize integrated physical identity assessment (organoleptic, macroscopic, and microscopic evaluation) and physicochemical specifications (e.g., limits for foreign matter, moisture, ash, and extractives), supported by chromatographic estimation of curcumin to verify composition and help detect adulteration or exhausted material (Singh et al. 2021). Microbiological testing (yeasts and molds, coliforms, and absence of *Escherichia coli* and *Salmonella* spp.) supports microbial quality, yet market evaluations indicate products may still vary in curcumin content and contain impurities (Britto et al. 2020). Therefore, chemical controls for toxicity indicators and contaminants (heavy metals, pesticide residues, aflatoxins, lead, and residual solvents) are also needed to ensure identity, composition, and safety (Skiba et al. 2018; Britto et al. 2020; Singh et al. 2021). The formulation and quality attributes are critical because they differ across preparations and can be influenced by co-administered enhancers and delivery technologies (Hegde et al. 2023).

Turmeric- and curcuminoid-containing dietary supplements should be prepared using current Good Manufacturing Practice (cGMP) that includes botanical authentication, use of the appropriate plant part, and testing the final product for herbicides, pesticides, heavy metals, fungal toxins, microbiological analysis and absence of mycotoxins, and concentrations of active constituents. Botanical authentication and testing of turmeric for adulteration have been carried out using liquid chromatography-mass spectrometry (LC-MS) (Ratnasekhar et al. 2025; Wei et al. 2025) and Fourier Transformation-Near Infrared spectroscopy (Guo et al. 2025). Testing of active constituents and contaminants in botanical dietary supplements can be guided by general pharmacopeial quality control frameworks and standard scientific methods, which address contaminants and impurities at the ingredient or product category level (Sarma et al. 2023; Matos et al. 2024; de et al. 2025). Multiple analytical methods for the determination and standardization of different curcuminoids in turmeric rhizomes, powders, extracts, and finished food or nutraceutical products have been reported, including spectrophotometric, chromatographic, capillary electrophoresis, and hyphenated mass spectrometric techniques (Kotra et al. 2019).

## Method

The USP DSAEL EC conducted a safety evaluation for turmeric and curcuminoids in a manner similar to that described in the USP Guideline for the Admission of Dietary Supplement Ingredients to the *USP–NF* Monograph Development Process, incorporating a comprehensive review of clinical and non‑clinical (toxicology) studies, clinical case reports, and adverse event (AE) data from multiple databases (USP 2009). Other important components were the presence of other pharmacopeial monographs and regulatory standards, chemistry and identity of materials of interest, potential adulterants and contaminants, intended uses, typical intake levels, and potential interactions.

The review was conducted as a structured safety evaluation and narrative synthesis of turmeric (*C. longa*) and curcuminoids, integrating evidence from (i) biomedical literature, (ii) published case reports/case series, (iii) public-facing spontaneous AE reporting databases, and (iv) regulatory and product-label sources to characterize potential hepatic safety signals across contemporary formulations and exposure contexts. For the biomedical literature component, an exploratory search was performed in PubMed® (National Center for Biotechnology Information, NCBI; https://pubmed.ncbi.nlm.nih.gov/) on 18 September 2025, a free resource that supports searching and retrieval of biomedical and life-sciences literature and contains more than 40 million citations and abstracts.

The PubMed^®^ search (1975–2025) using ‘turmeric’, ‘*Curcuma*’, and ‘curcumin’ retrieved >600 clinical-study records; therefore, clinical safety data synthesis prioritized higher-level evidence (systematic reviews, safety reviews, umbrella reviews, meta-analyses) identified *via* publication-type filters and Boolean safety strings (‘*Curcuma longa*’ OR turmeric OR curcumin OR curcuminoid*) AND (systematic review OR meta-analysis OR umbrella review OR safety review) AND (safety OR adverse event* OR toxicity OR hepatotoxic* OR ‘liver injury’ OR DILI OR HILI), with optional restriction to Humans and/or English-language records. Titles/abstracts were screened for relevance and full texts were reviewed as needed to confirm eligibility and extract safety outcomes; only review-type publications containing clinical safety information (for example, adverse events, tolerability, withdrawals due to adverse events, or safety laboratory outcomes) were included; individual clinical trials were not systematically extracted unless they provided safety details not captured in eligible reviews.

Non-clinical toxicology evidence was identified by expanding the ingredient search (e.g., ‘turmeric’, ‘curcumin’, and/or ‘*Curcuma longa*’) to include non-clinical concepts (e.g., animals, rats, mice, *in vitro*, acute, subacute, subchronic, repeated dose, genotoxicity, carcinogenicity, and reproductive/developmental toxicity). Eligible primary studies were retrieved through targeted searching and citation chaining from regulatory assessments, comprehensive reviews, and technical reports. Included study types comprised acute oral toxicity studies and repeat-dose dietary or gavage studies (including 14-day, 28-day, and 90-day/13-week designs). Long-term rodent studies, genetic toxicology batteries, and reproductive/developmental toxicity studies were also included. Pharmacokinetic and interaction evidence was retrieved using targeted terms related to disposition (for example, pharmacokinetics, bioavailability, absorption, metabolism, glucuronidation, sulfation, distribution, and excretion) and interaction mechanisms (for example, CYP, cytochrome P450, P-glycoprotein/P-gp, and transporter). Inclusion focused on human or animal pharmacokinetic studies and *in vitro* metabolism or transporter studies. Clinical or preclinical interaction studies were included when they reported effects on drug-metabolizing enzymes or transporters, or when they described clinically relevant interaction signals.

Exclusion criteria were: efficacy-only publications without extractable safety, toxicology, pharmacokinetic, or interaction outcomes; duplicate records; reports lacking sufficient test-article identification, dose/exposure description, or study-design detail to support interpretation (unless repeatedly cited in regulatory assessments and included with limitations). Comprehensive capture of all preclinical or clinical pharmacokinetic investigations was not feasible due to the rapidly expanding range of turmeric/curcuminoid products and delivery systems; therefore, pharmacokinetic evidence was integrated selectively, prioritizing foundational studies and those repeatedly cited in regulatory evaluations or directly informative for interpreting safety and interaction findings.

To complement clinical and non-clinical literature with real-world signal detection, searches were conducted on 29 September 2025 in multiple public-facing AE reporting systems using database-appropriate descriptors (‘turmeric’, ‘curcumin’, and/or ‘*Curcuma longa*’), recognizing differences in coding and search functionality across systems. AE database outputs were reviewed descriptively to identify the types of reported reactions and the presence of hepatobiliary event terms, but were interpreted strictly as hypothesis-generating because spontaneous reports are frequently incomplete, may include duplicates, cannot be used to estimate incidence or comparative risk, and do not establish causality. Published case reports and case series of clinically apparent liver injury temporally associated with turmeric/curcuminoid products were identified through bibliographic searching and reference-list screening and were summarized qualitatively with extraction of patient characteristics, product/formulation details (including co-ingredients when reported), latency, pattern of liver injury, dechallenge/rechallenge information, co-medications and other confounders, severity and seriousness, and outcomes (including hospitalization and acute liver failure).

Finally, regulatory reviews, safety advisories, and labeling actions, along with product-label information from online label repositories, were reviewed to capture risk communications and liver-related precautionary statements relevant to turmeric/curcuminoid-containing products. Evidence across streams was synthesized qualitatively, emphasizing clinically characterized hepatic outcomes and their relationship to formulation and exposure context; efficacy was not systematically evaluated except where efficacy studies contained safety-relevant information.

## Results and discussion

### Absorption, distribution, metabolism, and excretion (ADME)/pharmacokinetics

Turmeric curcuminoids exhibit very low oral absorption, extremely poor aqueous solubility, rapid phase I reduction and phase II conjugation, and fast elimination, which together yield very low systemic concentrations even after high oral doses up to 12 g (Fança-Berthon et al. 2021; EFSA 2010). Curcumin undergoes keto-enol tautomerism, with the keto form predominating in acidic conditions where it is largely water insoluble, while the enol form offers greater solubility but becomes unstable above pH 7. This pH dependent tautomerism collectively limits solubility and contributes to its poor oral bioavailability (Tønnesen and Karlsen 1985; FAO/WHO JECFA 2004). Curcumin’s biopharmaceutic behavior is consistent with Biopharmaceutics Drug Disposition Classification System (BDDCS) Class II like characteristics due to low solubility, instability in solution, and rapid metabolism leading to poor systemic availability (Shugarts and Benet 2009; McClements et al. 2015; Gerk 2021; Truzzi et al. 2021).

At acidic and neutral pH, water solubility is very low, limiting dissolution and absorption from standard turmeric or curcumin extracts used in most products (FAO/WHO JECFA 2004; Nelson et al. 2017). Co-administration of curcuminoids with lipid-rich food matrices (i.e., taken with food improves solubilization and increases systemic exposure of total curcuminoids, while plasma concentrations of unconjugated (free) curcumin remain minimal). In a randomized crossover trial, delivering the same colloidal turmeric extract in oat ‘milk’ or other food matrices raised dose normalized area under the curve (AUC)_0–24 h_ and maximum concentration (Cmax) for total curcuminoids versus capsules, with the lipid containing matrix giving the largest gains (AUC +76%, Cmax +105%) (Schönenberger et al. 2025). Mechanistically, milk/lipid carriers enhance curcuminoid dissolution/solubilization compared with water, supporting the fed-state effect (Kotha et al. 2022). Consistently, crossover pharmacokinetic studies show that enhanced formulations increase total (predominantly conjugated) curcuminoids, yet free curcumin remains approximately 1% of plasma curcuminoids with no meaningful rise in its AUC or Cmax (Fança-Berthon et al. 2021; Stohs et al. 2018).

In rats administered 1 g/kg body weight (bw) of curcumin orally, approximately 75% of the dose was excreted in feces, with only trace amounts appearing in urine. Plasma and bile concentrations were negligible, and blood concentrations remained below 5 μg/mL, indicating poor gut absorption (Wahlström and Blennow 1978; Ravindranath and Chandrasekhara 1980; EMA 2018a). *In vivo* in rats and mice, and in suspensions of human and rat hepatocytes, curcumin undergoes O-conjugation with glucuronic acid, and sulfate, and bioreduction to tetrahydrocurcumin, hexahydrocurcumin, and hexahydrocurcuminol, with LC-MS identifying glucuronic acid and sulfate conjugates as predominant plasma metabolites peaking at approximately 1 h (Asai and Miyazawa 2000; Ireson et al. 2002; EMA 2018a). Curcumin can be metabolized by a nicotinamide adenine dinucleotide phosphate (NADPH)‑dependent curcumin/dihydrocurcumin reductase in intestinal microbiota, producing dihydrocurcumin (DHC) and tetrahydrocurcumin (THC). THC, but not curcumin, has been reported to accumulate in rat tissues, indicating that gut microbiota may play an important role in curcuminoid metabolism and bioavailability (Fança-Berthon et al. 2021).

Most curcumin conjugation occurs in the intestine, where UGT1A8 and UGT1A10, along with UGT1A1, mediate phenolic glucuronidation, and sulfation is catalyzed by SULT1A1 and SULT1A3. The liver provides additional conjugation for the small fraction that escapes intestinal metabolism. These findings are based on *in vitro* studies using human intestinal and hepatic microsomes and recombinant enzymes, as well as *ex vivo* rat gut sac experiments (Ireson et al. 2002; Hoehle et al. 2007). Ireson et al. (2002) provided evidence from intestinal/hepatic fraction assays and rat studies indicating that, after oral dosing, metabolites entering the portal circulation are predominantly conjugated (e.g., glucuronides and sulfates), suggesting significant intestinal transformation before systemic circulation. Similarly, Ozawa et al. (2017) reported *in vivo* rat data showing that portal blood was enriched in conjugated forms compared to systemic blood, reinforcing the role of intestinal and hepatic first-pass metabolism in curcumin disposition.

Human studies consistently show poor systemic availability due to poor absorption and extensive first-pass metabolism, with most curcumin excreted unchanged in feces and negligible urinary excretion even at very high doses (EFSA 2010). Curcuma extract proprietary capsules prepared from *Curcuma spp.* (150 mg from *Curcuma domestica* and 50 mg from *Curcuma xanthorrhiza*) were given to patients with advanced colorectal cancer, providing doses equivalent to 36, 72, 108, 144, or 180 mg of curcumin. Neither curcumin nor its conjugates or reduced metabolites were detected in plasma or urine for up to 29 days. Curcumin was found in feces of all patients and curcumin sulfate was detected in feces of one patient at the highest dose (Sharma et al. 2001; EFSA 2010). In a separate study of 25 patients at high risk of malignancy, curcumin was administered at 500 mg/day and escalated stepwise up to 8,000 mg/day over a 3-month period. Dose escalation beyond 8,000 mg/day was not feasible because the tablet bulk was unacceptable to patients. Serum curcumin concentrations peaked 1–2 h after administration of 4,000–8,000 mg and declined within 12 h, with barely detectable levels at doses of 500–2,000 mg. Urinary curcumin was undetectable, and repeat pharmacokinetic sampling after more than one month of dosing in two patients showed no accumulation or time dependent changes (Cheng et al. 2001; EFSA 2010).

In healthy volunteers given oral curcumin at doses of 1.0 and 10 mg/kg bw, plasma curcumin concentrations were rarely above the limit of quantification despite pg/mL sensitivity, reaffirming low systemic exposure (Tullberg et al. 2004; EFSA 2010). Clinical pharmacokinetic studies consistently show that systemic circulation contains predominantly conjugated curcumin metabolites, mainly glucuronides and sulfates, while free curcumin is rarely detected. In a dose escalation trial, Lao et al. (2006) administered up to 12 g of curcumin to healthy volunteers and found negligible concentrations of unconjugated curcumin in plasma, detectable only at the highest doses. Vareed et al. (2008) confirmed this pattern in human subjects, reporting that glucuronide and sulfate conjugates account for nearly all circulating curcuminoids, underscoring the extensive presystemic metabolism and very low systemic availability of free curcumin.

Formulations such as liposomes, nanoparticles, micelles, phytosomes, and adjuvant combinations are marketed with ‘improved bioavailability’ claims, although few have been clinically tested at realistic consumer doses compared with standard extracts and the results are mixed (Fança-Berthon et al. 2021). In a randomized crossover study involving 23 healthy adults, a single 500 mg curcuminoid dose was administered as native powder, micronized powder, or liquid micelles. Compared with native curcumin, the plasma AUC for total curcuminoids was higher by 14-fold in women, 5-fold in men, and 9-fold overall with the micronized formulation, and higher by 277-fold in women, 114-fold in men, and 185-fold overall with liquid micelles (Schiborr et al. 2014). Women exhibited higher plasma AUCs than did men for all three curcuminoids except native curcumin. The plausible explanation provided by the authors for the sex-based differences in absorption is that men may have a higher expression of hepatic drug efflux transporters (e.g., P-glycoprotein) and metabolizing enzymes (e.g., glucuronosyltransferases and sulfotransferases), potentially leading to faster clearance of curcuminoids. In addition, differences in body weight and body composition may influence peak concentrations and distribution kinetics. The serum lipid concentrations, and liver and kidney function biomarkers remained within normal ranges throughout the study. In conclusion, this study demonstrates that curcumin bioavailability may be significantly enhanced through formulation strategies and highlights notable sex-based differences in curcumin absorption (Schiborr et al. 2014).

Another randomized, open-label, crossover trial involving 30 healthy adults compared five turmeric formulations: standard extract, micellar, piperine -combination, phytosome, and colloidal suspension) (Fança-Berthon et al. 2021). This trial uniquely compared marketed turmeric/curcuminoid formulations at their recommended daily doses (for consumer relevant findings). Plasma analysis over 24 h showed high variability, with conjugated metabolites (sulfates > glucuronides) dominating and unconjugated curcuminoids accounting for only approximately 1%. Overall AUC_0–24 h_ of unconjugated curcumin did not differ significantly among formulations; however, after dose normalization the colloidal suspension showed higher unconjugated curcumin exposure than the standard extract and the piperine-curcuminoid combination. No formulation–sex interaction was observed for AUC_0–24 h_ of total curcuminoids, but a formulation–sex interaction was detected for Cmax of total curcuminoids and for both Cmax and time to achieve Cmax (Tmax) of total curcumin metabolites and hexahydrocurcumin glucuronide. This study indicates that sex did not meaningfully affect the extent of exposure, while modest differences in absorption rate influenced peak concentration and time to peak. The authors noted that Schiborr et al. (2014) administered an unusually high dose of the micellar formulation alongside a much lower comparator dose, which resulted in an exaggerated approximately185-fold difference in relative plasma curcumin (Fança-Berthon et al. 2021). With evaluation of same micellar product at consumer‑recommended doses (micelles 1,000 mg vs. standard extract 1,500 mg), the advantage in unconjugated curcumin exposure was reduced to approximately 35.5‑fold, illustrating how dose bias can inflate apparent bioavailability estimates and why label‑recommended dosing provides a more realistic assessment (Fança-Berthon et al. 2021).

An older human study reported that co-administration of 20 mg piperine with 2 g curcumin markedly increased apparent systemic curcumin after oral dosing (Shoba et al. 1998). Later studies distinguishing unconjugated from conjugated forms did not confirm a benefit with piperine on free (unconjugated) curcumin. With modern liquid chromatography–tandem mass spectrometry (LC–MS/MS) analytical methods, piperine containing products show little to no meaningful impact on free curcumin bioavailability and do not consistently raise total curcuminoid exposure, compared to standard extracts (Fança-Berthon et al. 2021; Kroon et al. 2025). A real-world cohort study using validated high-performance liquid chromatography–tandem mass spectrometry similarly found no increase in plasma concentrations of free curcumin with adjuvants such as piperine among 47 users of curcumin products (Kroon et al. 2023). Collectively, these findings indicate that the piperine quantities typically present in commercial products (approximately 5–20 mg/dose) are insufficient to produce significant pharmacokinetic changes in either free curcumin or its conjugated metabolites.

Whether curcumin conjugates are hepatotoxic is unresolved, with regulatory and reviews emphasizing uncertainty and rare, inconclusive signals from case reports, including high-bioavailability products. Regulatory and review sources note that advanced formulations and adjuvants can raise measured exposure by multiple folds, although the clinical significance is limited because the systemic profile remains dominated by conjugates (EFSA 2010; Nelson et al. 2017; TGA 2023a; UK COT 2024). Genetic polymorphisms in UDP-glucuronosyltransferases (UGTs) and sulfotransferases (SULTs) could theoretically influence curcumin metabolism; however, functional redundancy among isoforms generally compensates for variability in phenolic substrate processing (Badée et al. 2019; Gamage et al. 2006; Hoehle et al. 2007).

Local β‑glucuronidase (GUSB) in bone marrow can hydrolyze curcumin‑glucuronide, regenerating aglycone curcumin; and at inflamed sites, neutrophil‑derived β‑glucuronidase deconjugates phenolic glucuronides, together supporting site‑specific regeneration and potentially higher local free curcumin despite low systemic parent concentrations (Kunihiro et al. 2019; Bartholomé et al. 2010). However, the clinical significance of tissue-level deconjugation remains unproven. Authoritative reviews conclude that translation into clear human benefit or risk has not yet been established (Bartholomé et al. 2010; Nelson et al. 2017). However, conjugated metabolites are not necessarily inert, and drug precedents such as morphine-6-glucuronide show that conjugates can retain pharmacological activity (Lötsch and Geisslinger 2001; van Dorp et al. 2006; Klimas and Mikus 2014).

A recent study systematically assessed disintegration, dissolution, and biorelevant bioaccessibility of curcuminoids and (S)-ar-turmerone from eight commercially available turmeric dietary supplements spanning diverse formulation strategies using USP-aligned methods using in fasted- and fed-state simulated gastric and intestinal media. Bioaccessible concentrations, quantities, and dose fractions were quantified after 3 h. Across products, overall dissolution was poor, with no formulation exceeding 40% total release; release was lowest in fasted-state media and increased only modestly in fed-state gastric conditions, consistent with lipid-assisted solubilization. Higher curcuminoid load per capsule tended to yield higher absolute bioaccessible concentrations despite low release efficiency, whereas a phytosome product showed comparatively better release despite a lower dose, and several products marketed as ‘enhanced’ performed poorly due to incomplete disintegration and low bioaccessibility. The findings imply that observed bioaccessible levels are jointly governed by dosage-form performance and dose loading, and that plasma ‘bioavailability’ metrics dominated by conjugated metabolites may not reliably track formulation performance or meaningful delivery of free curcumin, particularly given curcumin’s intrinsic challenges (poor solubility, instability, variable dosage-form behavior, and extensive first-pass metabolism). Practically, the data suggest administration with food is more likely to improve bioaccessibility than fasted dosing, yet labeling recommendations are inconsistent, potentially limiting real-world exposure when products are taken with water alone. The study also highlights broader interpretive issues, including the possibility that conjugated metabolites could still contribute to pharmacology *via* local deconjugation and that non-curcuminoid constituents such as (S)-ar-turmerone add complexity not captured by curcuminoid-only dissolution readouts, reinforcing that superior *in vitro* release is a prerequisite but not a guarantee of enhanced systemic exposure and that bioavailability claims for commercial turmeric supplements warrant cautious interpretation (Gurley et al. 2026).

**Figure 1.**
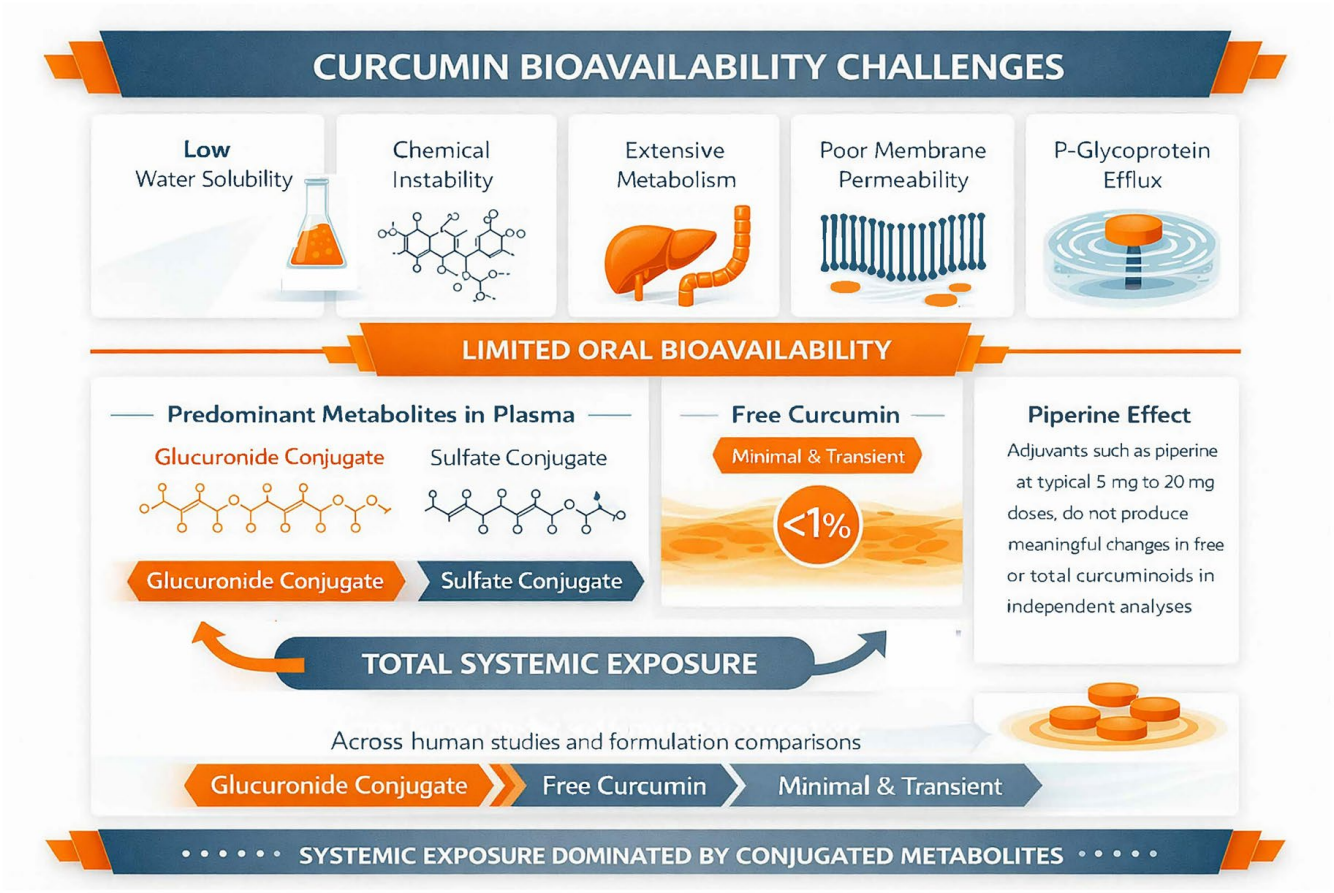
Overview of the major factors limiting oral curcumin bioavailability, showing that systemic exposure in humans is dominated by glucuronide and sulfate conjugates, while free curcumin remains minimal and transient despite formulation strategies or adjuvants such as piperine.

Overall, from pharmaceutical and pharmacokinetic perspectives, curcumin combines low solubility, chemical instability, extensive intestinal and hepatic metabolism, low membrane permeability, and active efflux *via* P-glycoprotein, which jointly limit oral bioavailability of the parent compound. Across human studies and formulation comparisons, systemic exposure is overwhelmingly due to glucuronic acid and sulfate conjugates, and free curcumin remains minimal (Figure 1) and transient even when total exposure increases with enhanced delivery or fed-state conditions (Lao et al. 2006; Vareed et al. 2008; Stohs et al. 2018; Fança-Berthon et al. 2021; UK COT 2024). Consequently, consumer-relevant formulation differences are modest for total systemic exposure when doses are matched, with limited and inconsistent improvements in unconjugated curcumin. Adjuvants such as piperine at typical 5 mg to 20 mg doses do not produce meaningful changes in free or total curcuminoids in independent analyses (Fança-Berthon et al. 2021; Kroon et al. 2023; 2025).

### Potential drug interactions

Curcuminoid extracts inhibit multiple human Cytochrome P450 (CYP) isoforms *in vitro*, with potent effects on CYP2C19, CYP2B6, and CYP2C9 and moderate effects on CYP3A, whereas CYP1A2 and CYP2D6 are weakly affected. For CYP2C19, CYP2B6, CYP2C9, and CYP3A, the IC_50_ values are in the low‑to‑mid micromolar range. These data support a mechanistic basis for herb‑drug interactions. However, a short 2‑day curcuminoid plus piperine regimen did not produce clinically meaningful interactions with CYP3A or CYP2C9 probe drugs in healthy volunteers (Volak et al. 2008; 2013; Mashayekhi-Sardoo et al. 2021). Curcumin also inhibits major human glutathione S-transferases (GSTA1-1, GSTM1-1, GSTP1-1) and forms glutathionyl conjugates catalyzed by GSTP1-1, consistent with phase II modulation at micromolar concentrations (Appiah-Opong et al. 2009; Awasthi et al. 2000).

In male Sprague–Dawley rats, oral curcumin (60 mg/kg/day for 4 days) downregulated intestinal P-gp (−49%) and CYP3A (−42%), while upregulating hepatic P-gp (+144%) and CYP3A (+91%) and renal CYP3A (+41%), demonstrating tissue-specific regulation and predicting increased oral exposure to P-gp/CYP3A substrates (Zhang et al. 2007). Consistent with these changes, curcumin pretreatment increased celiprolol Cmax (1.9-fold) and AUC_0–8 h_ (1.6-fold) and reduced oral clearance (−22%), consistent with enhanced absorption *via* intestinal P-gp suppression. Midazolam AUC_0–4 h_ and total AUC increased 2.6- and 3.8-fold, with a 75% reduction in clearance, consistent with reduced intestinal CYP3A-mediated first-pass metabolism (Zhang et al. 2007). Another study in rats showed turmeric juice (and grapefruit/ginger) significantly increased tacrolimus exposure, supporting food–drug interactions *via* CYP3A/P-gp inhibition relevant to calcineurin inhibitors (Egashira et al. 2012).

Clinical evidence includes two cases involving patients taking tacrolimus: One liver transplant recipient developed acute kidney injury and markedly elevated tacrolimus concentrations after consuming approximately 15 spoonfuls (about 75 g/day) of turmeric daily for 10 days, attributed to CYP3A inhibition (Nayeri et al. 2017). In contrast, a case report in a renal transplant patient consuming turmeric (10 g/day for 4 days) showed no significant effect, suggesting excessive intake may increase tacrolimus exposure, while routine dietary amounts appear safe (Boissiere et al. 2023). A 56‑year‑old woman taking fluindione experienced an elevation in international normalized ratio (INR) from 2–3 to 6.5 after consuming turmeric infusions (2.5 g/day for 5 days), which resolved upon discontinuation, indicating a potential interaction between turmeric and vitamin K antagonists (Daveluy et al. 2014). Similarly, some warfarin-treated patients have presented with marked INR elevation (>10) after initiating turmeric/curcumin supplements, prompting safety warnings that concentrated preparations of turmeric/curcumin may potentiate the activity of warfarin (Medsafe NZ 2018).

Across *in vitro* studies, with supportive preclinical data, curcumin and its glucuronide metabolite (curcumin‑O‑glucuronide) interact with OATP1B1/OATP1B3 as substrates and inhibitors, whereas evidence for curcumin‑O‑sulfate at these transporters is indirect; collectively, these interactions may influence drug pharmacokinetics (Sun et al. 2016; Zhou et al. 2017). In intestinal LS180 cells, curcumin inhibits P-gp activity independent of formulation, which could increase absorption of P-gp substrates; however, human crossover data show subject-dependent variability in digoxin disposition (Flory et al. 2021; Koonrungsesomboon et al. 2021). Despite *in vitro* inhibition of CYPs and conjugation enzymes, a randomized, placebo-controlled crossover involving healthy volunteers found that 4 g of curcuminoids plus 24 mg of piperine over 2 days did not significantly alter midazolam (CYP3A), flurbiprofen (CYP2C9), or paracetamol (UGT/SULT) pharmacokinetics, suggesting no clinically meaningful interaction with short-term use (Volak et al. 2013).

Most *in vitro* studies evaluate free (unconjugated) curcumin under solubilizing conditions at micromolar concentrations, but these conditions are not physiologically relevant because systemic exposure to unconjugated curcumin is negligible, even with high oral doses or ‘enhanced bioavailability’ formulations (Bahramsoltani et al. 2017; Nelson et al. 2017; UK COT 2024; Kroon et al. 2025). Accurate quantitation of curcumin is challenging since plasma contains diglucuronic acid, disulfate, and mixed sulfate–glucuronic acid as well as monoglucuronic acid and monosulfate conjugates. Hydrolysis with β-glucuronidase (GUSB) alone underestimates total curcumin because sulfate-containing species remain intact unless sulfatase or direct LC–MS/MS measurement is employed (Trontelj 2012; Luis et al. 2020). Consequently, most published herb–drug interaction studies use parent compounds (curcumin/curcuminoids), not their conjugates, limiting translational relevance for systemic tissues where conjugates predominate (Vareed et al. 2008; Volak et al. 2008; Volak et al. 2013; Bahramsoltani et al. 2017; Mashayekhi-Sardoo et al. 2021). The liver is exposed mainly to glucuronic acid/sulfate conjugates, with unconjugated plasma concentrations typically near or below detection in human studies (Nelson et al. 2017; Kroon et al. 2025). Glucuronides are also important for toxicology and pharmacokinetics of many drugs and xenobiotics. Although glucuronides often lack pharmacological activity, deconjugation can regenerate active aglycones and may pose an environmental burden (Luis et al. 2020). It has been noted that GUSB is expressed in hepatocytes and hepatic macrophages (Kupffer cells), though its distribution and activity *in vivo* depend on the inflammatory milieu (de Graaf et al. 2002; Li et al. 2022; Human Protein Atlas 2026). The extent of *in situ* deconjugation of curcuminoid conjugates within hepatic tissue remains unclear, and current evidence does not establish whether local GUSB activity regenerates free aglycone at clinically relevant exposures (Luis et al. 2020). Notably, GUSB activity is enriched at inflamed or necrotic sites (e.g., tumor microenvironments), a principle exploited in prodrug strategies. By analogy, preexisting hepatic injury or inflammation with increased macrophage/neutrophil activity could enhance local deconjugation, potentially exposing adjacent hepatocytes to free aglycone; a possibility but unproven mechanism warranting targeted study (de Graaf et al. 2002; Li et al. 2023). Overall, intestinal first-pass interactions (CYP3A4/P-gp) remain the most defensible mechanism for curcuminoid herb–drug effects, whereas systemic hepatic interactions inferred from parent compound *in vitro* data should be interpreted cautiously unless conjugate pharmacology or local deconjugation is demonstrated in human tissues.

Curcuminoid-related interaction potential should be interpreted in the context of limited systemic exposure after oral intake, because human pharmacokinetic studies generally show that circulating free (unconjugated) curcumin is often low or not detectable (Vareed et al. 2008; Volak et al. 2013; Fança-Berthon et al. 2021). *In vitro*, curcuminoids (and piperine in some preparations) can inhibit multiple drug-metabolizing enzymes (including CYPs, UGTs, and SULTs) at micromolar concentrations, supporting biological plausibility for interactions under certain exposure conditions (Volak et al. 2008). However, a controlled crossover study in healthy volunteers found that short-term administration of a high-dose curcuminoid–piperine preparation did not meaningfully alter the pharmacokinetics of probe substrates for CYP3A, CYP2C9, or acetaminophen conjugation pathways, while unconjugated curcuminoid concentrations remained below assay thresholds. (Volak et al. 2013). Preclinical work indicates that repeated curcumin dosing can modulate intestinal P-gp and intestinal CYP3A expression and increase exposure to orally administered probe substrates in rats, consistent with a plausible presystemic mechanism under repeated dosing scenarios (Zhang et al. 2007).

Recent case-based evidence highlights that signals are most relevant for narrow therapeutic index drugs and anticoagulants, including a structured hospital-pharmacy protocol in a renal transplant recipient showing negligible tacrolimus changes with short, controlled spice exposures, alongside reports of elevated INR with vitamin K antagonists and more recent bleeding-associated supratherapeutic INR with warfarin in the setting of turmeric supplement use (Daveluy et al. 2014; Boissiere et al. 2023; Stewart et al. 2025). Regulatory communications reinforce this distinction by cautioning that turmeric/curcumin-containing products may interact with warfarin while explicitly noting that the warning does not apply to turmeric used in food (NZ Medsafe 2018), and Health Canada has issued precautionary labeling actions for oral turmeric/curcuminoid-containing natural health products emphasizing stop use and medical consultation (including when medicines are used concomitantly) (Health Canada 2025).

### Toxicity in animal models and *in vitro*

Numerous acute toxicity studies in both mice and rats have been conducted with various extracts (solvent-based, aqueous, essential oils, oleoresins, and polysaccharide) of turmeric rhizome powder and proprietary forms of curcumin administered orally either by gavage or as feeding studies. Many of these studies included single doses as high as 5,000 mg/kg bw of test article with no mortality and few toxicity findings reported. The lack of significant findings and LD_50_ values > 5,000 mg/kg bw from these acute studies in rodents with turmeric and curcumin indicate a low order of toxicity.

Several repeated dose oral toxicity studies in rodents of 28- and 90-day durations have also been conducted with turmeric rhizome ethanol extract, curcuminoid-essential oil complexes, bisdemethoxycurcumin-enriched extracts, synthetic curcumin, and turmeric oleoresin. These studies also resulted in no treatment-related mortality or systemic toxicity at doses up to 1,000–2,000 mg/kg bw/day. There were isolated findings in some of these studies (e.g., gastric erosion at 100 mg/kg bw/day in rats and mild liver fatty degeneration at 250 mg/kg bw/day); but they were found to be reversible and not considered relevant. Thus, the No Observed Adverse Effect Levels (NOAELs) were all established as the highest doses tested.

Repeated dose studies have also been conducted in rodents in which hepatotoxicity was the toxicology endpoint of focus. Kandarkar et al. (1998), conducted a 14-day study in inbred female Swiss albino mice. Turmeric (0%, 0.2%, 1.0%, and 5.0%) or an ethanolic turmeric powder extract (0%, 0.05%, 0.25%) was added to the diet and fed to mice. Based on the histopathology evaluation of the liver: 0.2% turmeric resulted in coagulative necrosis in 3 of 6 mice; 1.0% turmeric caused degenerative changes, necrotic foci, and regenerative zones in the liver of 3 of 6 mice; and 5% turmeric caused necrotic changes in all mice (i.e., 6 of 6). Treatment with the 0.05% and 0.25% ethanolic turmeric powder extract resulted in similar hepatic alterations. Both turmeric (1.0% and 5.0%) and the ethanolic turmeric powder extract caused ultrastructural liver findings including clumped chromatin, vacuolated mitochondria with dense bodies, abundant glycogen, and prominent Golgi bodies. The 0.2% turmeric resulted in similar but less pronounced changes. The dose levels used in these studies were 200–5,000 times higher than the Acceptable Daily Intake (ADI) of 0–2.5 mg/kg, established by the Joint Food and Agriculture Organization (FAO) and the World Health Organization (WHO) Expert Committee on Food Additives (JECFA), and 8–200 times higher than the estimated human intake of 4 g/day.

Deshpande et al. (1998) also studied hepatotoxicity associated with turmeric (powdered rhizomes) and ethanolic turmeric extracts (98% curcumins). An initial 14‑day study was conducted in inbred female Swiss mice and Wistar rats receiving 1.0% and 5% turmeric or 0.05% and 0.25% ethanolic turmeric extract in diet; in a separate mouse‑only experiment, 0.01%, 0.1%, and 0.2% turmeric were also evaluated. There was no effect on body weight during the 14-day study in either mice or rats. Liver histopathology showed necrosis in mice treated with 5% turmeric (8 of 12 mice with regeneration, and 4 of 12 mice without regeneration). Similar effects were observed at 1% turmeric, and 0.05% and 0.25% ethanolic extract. No abnormalities were observed in rats. There was also spleen necrosis observed in mice at 5% turmeric and 0.05% and 0.25% ethanolic extract. These same authors did a follow-up 90-day toxicity study in both rodent species using 0 and 5% turmeric. Body weight gain was decreased in the 5% treatment group in both mice and rats. Necrosis was observed in 5 of 6 mice and 2 of 6 rats at the 5% dose levels. Surprisingly, serum biochemical markers including serum glutamic oxaloacetic transaminase (SGOT) and serum glutamic pyruvic transaminase (SGPT), urea, creatinine, protein, albumin, and hemoglobin were normal in mice. An increase in SGOT, urea, albumin, and hemoglobin, and decrease in protein was observed in rats. The authors highlighted the unexpected findings of liver necrosis without SGOT/SGPT elevation and mild necrosis with increase in SGOT but not SGPT. It appeared from this study that mice may be more susceptible to turmeric-induced liver damage than are rats. Due to the high doses administered in this study compared to JECFA’s ADI (200–5,000 times) or human intake levels (50 times higher than 0.6 g/adult/day estimate), the authors concluded that the hepatotoxicity observed did not raise concern for normal dietary use but may warrant further mechanistic studies.

The National Toxicology Program (NTP) conducted 2-year bioassays in F344/N rats and B6C3F1 mice using turmeric oleoresin containing 79–85% curcumin (NTP, 1993). Doses of turmeric oleoresin were administered in the feed at 0, 2,000, 10,000, and 50,000 ppm (corresponding to approximate daily intakes of 80, 460, and 2,000 mg/kg/day in male rats and 90, 440, and 2,400 mg/kg/day in female rats; 220, 520, and 6,000 mg/kg/day in male mice; and 320, 1,620, and 8,400 mg/kg/day in female mice). At the conclusion of the study, male rats showed no evidence of carcinogenicity. There was an equivocal finding of a slight increase in clitoral gland adenomas in female rats as no clear dose-response relationship was observed. In mice of both sexes there was equivocal evidence of carcinogenicity. In male mice there was an increased incidence of hepatocellular adenomas at 10,000 ppm and small intestine carcinomas at lower doses. Female mice had a slight increase in hepatocellular adenomas. The NTP Technical Reports Review Subcommittee considered upgrading the evidence in mice to ‘some evidence’, but ultimately retained the ‘equivocal evidence’ classification. It was noted that there were limitations in the studies including the lack of testing with pure curcumin, uncertainty about the biological activity of non-curcumin components in turmeric oleoresin, and the lack of robust data on metabolism and disposition of turmeric and curcumin in the species tested (NTP 1993).

Although turmeric itself has not been tested in a well-conducted developmental and reproductive toxicity study, curcumin and curcuminoids have been studied according to Organization for Economic Co-operation and Development (OECD) guidelines (Ganiger et al. 2007; Majeed et al. 2019, 2023). Curcumin (≥95% purity) was tested in a two-generation reproductive toxicity study in rats at doses of 0 (control), 1500, 3000, and 10,000 ppm in the diet. The authors concluded that no adverse effects were observed in fertility, gestation, or pup development across two generations. In the JECFA review of this study, a slight reduction in F2 pup pre-weaning body weight at the highest dose was considered as adverse and the No Observed Effect Level (NOEL) was set at 250–320 mg/kg bw/day, which was the mid-dose level. As a result, JECFA established an ADI of 0–3 mg/kg bw based on the NOEL and this was later supported by European Food Safety Authority (EFSA) (JECFA 2004; EFSA 2010).

A bisdemethoxycurcumin‑enriched *C. longa* extract (AC3^®^) was tested according to OECD 421 guidelines in Wistar rats. The test substance was standardized to ≥85% total curcuminoids (bisdemethoxycurcumin content of 30–35%). Doses tested were 0, 125, 250, and 500 mg/kg bw/day. A NOAEL of 500 mg/kg bw/day was based on no observed adverse effects at any of the tested doses (Majeed et al. 2023).

Another OECD 421 study was conducted with tetrahydrocurcuminoids (95.4% purity from *C. longa*) in Wistar rats at doses of 0, 100, 200, and 400 mg/kg bw/day *via* oral gavage (Majeed et al. 2019). Maternal body weights were significantly reduced by 6.6–8.6% in the high-dose group during gestation. One dam treated in the 100 mg/kg bw/day group had 9 out of 10 pups with tail deformities which was considered an isolated occurrence. Thus, no significant findings in any reproductive parameters were identified by the study authors. The EFSA review panel evaluated this same study and identified post-implantation loss of 30% versus 15.2% in the control group, a live birth index of 83.1% versus 95.8% in the control group, and 14 dead pups from dams versus 4 dead pups in the control group. Although not statistically significant, EFSA considered these findings biologically relevant and set the NOAEL at 200 mg/kg bw/day (versus the 400 mg/kg bw/day proposed by the study authors) (EFSA 2021).

There have been other studies conducted that have identified potential reproductive toxicity findings and antifertility effects in rats treated with turmeric or curcumin. These studies were not conducted according to any standard guidelines and in some cases the test articles were not characterized, or doses were not specified (Chen et al. 2010; Chen and Chan 2012, Hashem et al. 2011; Huang et al. 2013; Maiti et al. 2021; Busman et al. 2022). Curcumin given orally has potentially shown antifertility effects in female rats including antigonadotropic, antiestrogenic, anti-implantation, or abortifacient effects at doses between 25- and 100 mg/kg bw/day (Maiti et al. 2021). However, due to the weaknesses of the study designs as mentioned previously, little can be gleaned from these findings.

Numerous *in vitro* and *in vivo* genotoxicity studies have been conducted with turmeric, and curcumin and other curcuminoids (Giri et al. 1990; Vijayalaxmi 1980; Jensen 1982; Jain et al. 1987; Liju et al. 2013; Aggarwal et al. 2016; Damarla et al. 2018; Ravikumar et al. 2018; Do and Kwon 2022; Dziwenka et al. 2022; Nirvanashetty et al. 2022; Majeed et al. 2023, NTP 1993). Most of these studies have reported negative findings; however, there have been some positive results in both *in vitro* and *in vivo* assays. The EFSA review panel concluded that although curcumin showed genotoxicity in some studies, it was not carcinogenic in long-term NTP studies (see above NTP 2-year bioassays).

**Figure 2.**
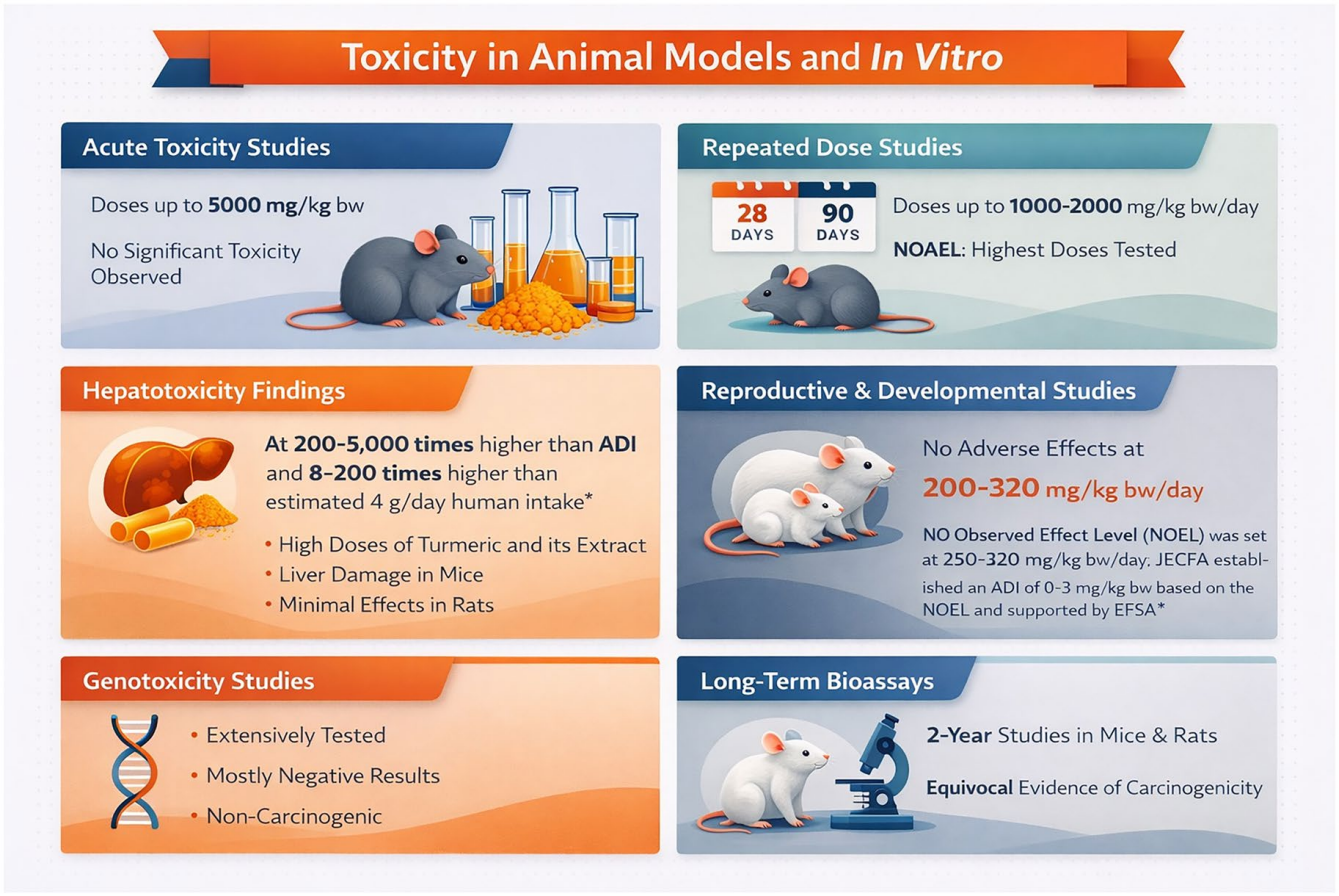
Summary of nonclinical toxicity evidence for curcumin and turmeric preparations in animal models and *in vitro* systems. * values marked with an asterisk are derived from regulatory assessments or literature-based estimates (EFSA/JECFA and estimated human intake).

In summary, hepatoxicity was observed in repeated dose rodent studies but only at dose levels far in excess of estimated dietary consumption of turmeric. Both turmeric and curcumin have been exhaustively tested in various *in vitro* and *in vivo* genotoxicity studies. Although some studies did result in positive findings, various authoritative bodies have concluded that turmeric and curcumin are non-genotoxic and non-carcinogenic. Some signals of impaired fertility or effects on fetal development in various experimental models have been noted, but these were only found in non-guideline studies. In a well-conducted reproductive study, NOAEL was established at 200–320 mg/kg bw/day (Figure 2).

Across regulatory evaluations, the non-clinical evidence base for turmeric and curcumin is centered on guideline-compliant genotoxicity studies, repeated-dose oral toxicity studies, reproductive toxicity studies that support ADI values, and long-term rodent bioassays conducted with turmeric oleoresin. These datasets are complemented by formulation-specific toxicity studies for commercial preparations, which are informative for the tested materials but are not necessarily generalizable across turmeric or curcuminoid products with different compositions or bioavailability characteristics (UK COT 2024; EFSA 2010).

Acute oral toxicity studies of turmeric- and curcumin-derived preparations in rodents generally indicate low acute toxicity and high tolerability at the highest doses tested (Dziwenka et al. 2022; Nirvanashetty et al. 2022; Majeed et al. 2023). In repeat-dose rodent studies, including subchronic feed and gavage studies, systemic toxicity is typically limited at commonly tested dose levels for characterized extracts and formulations. Formulation-specific toxicology studies reported in the recent literature provide additional context for well-characterized commercial preparations. For example, a 90-day oral gavage study of an oleoresin-based turmeric extract (CURCUGEN^®^) reported no adverse findings at the limit dose tested and supported a NOAEL at that dose under the study conditions (Nirvanashetty et al. 2022). Similarly, a Good Laboratory Practice (GLP)-aligned safety evaluation of a curcumin complex (CuminUP60^®^) reported negative results in the genotoxicity test battery described, high acute tolerability, and a subchronic NOAEL in rats for the tested formulation (Dziwenka et al. 2022). A bisdemethoxycurcumin-enriched extract (AC3^®^) has also been evaluated in acute, repeated-dose, reproductive/developmental, and genotoxicity study components under OECD/GLP principles, with no adverse findings reported at the tested dose levels (Majeed et al. 2023). These studies strengthen the evidence base for the specific preparations evaluated, but conclusions should remain linked to the tested materials and their characterization.

In NTP 13-week dietary studies of turmeric oleoresin (79–85% curcumin) in rats and mice, increased absolute and relative liver weights were reported at mid and high dietary concentrations, with no accompanying histopathologic changes. On this basis, JECFA and EFSA interpreted the liver weight findings as non-adverse at the exposure levels evaluated (NTP 1993; UK COT 2024). Within the animal toxicity studies summarized in recent regulatory reviews, the liver is repeatedly identified as a key organ of interest for turmeric and curcumin preparations, based on observations across multiple study types, including liver weight changes in the NTP feed studies and hepatic findings reported in certain high-exposure feeding studies using turmeric materials. Regulatory summaries also note that the interpretation of hepatic findings depends on study context, including dose levels, test material characterization, and consistency across species and study designs (UK COT 2024). Further, the TGA has noted that the non-clinical data include both studies reporting hepatotoxicity at high exposures and a broader body of studies showing minimal or no adverse findings, but emphasizes that idiosyncratic liver injury is difficult to model and replicate in animals, limiting the ability of non-clinical studies to exclude rare human outcomes (TGA 2023b).

Genotoxicity findings are largely negative *in vivo* and in bacterial assays, but mixed *in vitro* results have been reported for some preparations (including chromosomal aberrations and sister chromatid exchanges). Several studies are difficult to interpret because of incomplete information on curcumin purity or preparation details, leading JECFA to conclude that there was inadequate evidence of genotoxicity for curcumin (EMA 2018a; EFSA 2010). Long-term rodent data do not support a conclusion of carcinogenicity for curcumin under evaluated conditions. EFSA also discussed the NTP long-term findings and noted that statistically significant neoplastic findings were largely confined to benign lesions, lacked consistent dose-response, and were not consistent across sexes and species (EFSA 2010).

Nonclinical evidence summaries in major regulatory assessments support a food additive-based ADI of 0–3 mg/kg bw/day for curcumin, derived from a NOEL/NOAEL in a reproductive toxicity study. However, extrapolation from food additive-based assessments to supplement products may be inappropriate when synthetic or adjuvanted preparations alter toxicokinetic profiles. These reviews emphasize that safety conclusions should be anchored to well-characterized test materials and clearly defined exposure conditions, and that extrapolation to supplement products warrants caution when formulations alter systemic exposure and toxicokinetics, particularly for products designed to enhance bioavailability (EFSA 2010; UK COT 2024; TGA 2023b).

### Human toxicity

Human toxicity evidence for turmeric and curcuminoids was assembled from complementary sources, including PubMed^®^-indexed clinical publications, case reports and case series, and public-facing spontaneous AE reporting systems. The searches identified an extensive clinical literature evaluating turmeric- and curcuminoid-containing products across a broad range of formulations, doses, and treatment durations, with safety information variably reported across trials and synthesized in review-level publications. Post-marketing evidence sources provided additional insight into real-world reporting patterns, with spontaneous report data dominated by non-serious reactions (particularly gastrointestinal and hypersensitivity-type events) but also containing reports coded to hepatobiliary outcomes that warrant contextual evaluation. Published case reports and case series provide the most detailed clinical characterization of suspected liver injury, including latency, biochemical patterns, severity, and clinical course, although interpretation is limited by incomplete product characterization, co-exposures, and heterogeneity in reporting. Taken together, these evidence streams support an integrated perspective in which clinical trial data inform general tolerability under controlled conditions, spontaneous reports provide hypothesis-generating post-marketing signals, and case reports/series help define the clinical features of uncommon but clinically important events.

#### Clinical trials

A PubMed^®^ search using the terms ‘turmeric’ and ‘curcumin’, identified more than 600 results classified under the article type ‘clinical studies’ for the period 1975–2025. Summarizing all individual trials was not feasible for the USP DSAEL EC admission evaluation. Therefore, to ensure a scientifically robust and comprehensive synthesis of evidence, our evaluation included only systematic reviews, safety reviews, umbrella reviews, and meta-analyses. The justification for this approach was that these high-level evidence sources aggregate data from multiple clinical trials and apply rigorous quality assessments. Notably, PubMed^®^ lists over 150 such reviews, but only those that included clinical safety-related information were considered in the admission evaluation.

An umbrella review conducted included 25 meta-analyses of randomized controlled trials (RCTs). The study population included adults ≥18 years, both healthy and with conditions such as Type 2 diabetes (T2DM), nonalcoholic fatty liver disease (NAFLD), polycystic ovary syndrome (PCOS), rheumatoid arthritis (RA), ulcerative colitis, COVID-19, osteoarthritis, anxiety, or depression. Intervention treatments were oral curcumin/turmeric (50–6,000 mg/day) and duration of treatments ranged from 1 day to 12 months. Comparators varied from placebo to routine care and standard medications. There were no serious adverse events (AEs) reported. Mild AEs recorded included gastrointestinal (GI) discomfort, headache, rash, or dizziness. Liver and kidney function was assessed in 2 meta-analyses with no adverse changes recorded. The AMSTAR-2 (A MeaSurement Tool to Assess systematic Reviews 2) was used to assess quality of the studies included in the umbrella review and 76% were considered very low and 12% of moderate quality. The limitations included high heterogeneity (regarding participants, interventions, and assessment of outcomes), lack of protocol registration, English-language bias, reliance on secondary data, low study quality, and potential bias. The recommendations for the meta-analyses were to clearly justify inclusion of each study, list the excluded studies with reasons to reduce bias, and record funding sources to identify potential conflicts of interest (Xu et al. 2025).

Another umbrella review included 26 systematic reviews and meta-analyses (72 RCTs and 87 study arms). The population included was similar as that reported in Xu et al. (2025) but included subjects with chronic kidney disease and dyslipidemia. Interventions were categorized into three groups: 1) whole‑compound preparations (15 studies), typically providing 500–3000 mg/day of turmeric or *C. longa* powder or standardized turmeric; 2) curcumin‑extract formulations (35 studies), with doses ranging from 45 to 6000 mg/day; and 3) bioavailability‑enhanced products (37 studies), which used a variety of delivery technologies and dosing regimens. These enhanced formulations included curcumin 500–4500 mg combined with piperine 5–30 mg, nano‑curcumin 80–120 mg/day, phytosomal or phospholipid/liposomal forms 250–1000 mg/day, micellar curcumin 294–1000 mg/day, cyclodextrin‑complexed curcumin 1200 mg, lecithin‑formulated curcumin 400 mg, and amorphous‑dispersion curcumin 500 mg, reflecting the wide variability in enhanced‑absorption strategies across studies. The durations of treatment included 1–24 weeks and comparators were placebo or standard treatments with some exercise. No safety or adverse outcomes were mentioned across any of the studies included in this review. Liver function markers listed for some studies included alanine aminotransferase (ALT), aspartate aminotransferase (AST), gamma‑glutamyl transferase (GGT), and alkaline phosphatase (ALP) with no pooled results reported. The AMSTAR-2 was also used to assess quality of the studies included in the review and 4 were rated as high, 8 moderate, 10 low, and 4 critically low in quality. Thirty-three of 72 RCTs had a high risk of bias. The limitations identified across studies included high heterogeneity, variable formulations and doses, and inconsistent methodological rigor (Unhapipatpong et al. 2025).

Evidence was assessed from 6 systematic reviews and meta-analyses published between 2019 and 2022, each including 4 to 11 randomized controlled trials, evaluating curcumin supplementation in individuals with NAFLD confirmed by ultrasonography (Goodarzi et al. 2019; Jafarirad et al. 2019; Mansour-Ghanaei et al. 2019; Wei et al. 2019; White and Lee 2019; Yang et al. 2022). Adults (228–2,173 per review) aged 40–65 years were given an intervention of turmeric (2,000–3,000 mg/day) or curcumin (80–3,000 mg/day) for 4–24 weeks. The formulations tested included unformulated, lipidated, phospholipid-complexed, micellar, phytosomal, or nano-curcumin, or in combination with piperine. No serious AEs were reported across studies and there were significant reductions in liver enzymes (e.g., ALT and AST) especially with duration <12 weeks and doses ≥1,000 mg/day. There were no adverse changes in liver enzymes or other metabolic parameters assessed. Mild, non-serious adverse effects were recorded in some trials and included nausea, stomachache, abdominal pain, headache, rash, bruising, feeling cold, or GI discomfort. The limitations identified included small sample sizes; short follow-up durations; lack of gender-specific analysis; geographic restriction to Asian populations; missing data on parameters such as ALP, GGT, and monitoring of long-term outcomes (e.g., liver fibrosis).

Dehzad et al. (2023) performed a systematic review and dose-response meta-analysis with a focus on liver function using 31 RCTs and 1,948 participants aged 18–70 years. Subjects had similar conditions as previously mentioned but also included obesity, metabolic syndrome, osteoarthritis, beta-thalassemia, ulcerative colitis, cirrhosis, and alcoholism. Interventions involved 80–3,000 mg/day of turmeric, curcumin, curcuminoids, nano-curcumin, as well as high-absorption forms of each given over 4–24 weeks. There was no characterization of supplement purity or potential contamination. Liver enzyme outcomes were monitored using a weighted mean difference (WMD) and resulted in ALT WMD of −4.09 U/L; AST of −3.81 U/L; no significant effect on GGT (WMD of −12.78 U/L). No significant association between dose or duration and enzyme levels was identified (non-linear dose-response). A Grading of Recommendations Assessment, Development and Evaluation (GRADE) was used to rate the quality (or certainty) of evidence presented. The resulting GRADE was low quality for ALT and AST and very low quality for GGT.

Several RCTs that reported on specific findings were reviewed. For example, Rainey-Smith et al. (2016) conducted a 12-month, double-blind, placebo-controlled trial involving 160 cognitively healthy, community-dwelling older adults. Treatment comprised Biocurcumax^®^ (BCM-95^®^ CG, also known as Curcugreen^®^), a patented, highly bioavailable turmeric extract combining curcuminoids with oils of turmeric (e.g., ar-turmerone) which purportedly enhances absorption and increases bioavailability. BCM-95^®^ CG was administered at 1,500 mg/day, administered as 500 mg three times per day. Twenty-three participants (18 from the treatment group and 2 from the placebo group) withdrew due to AEs that were predominantly GI-related symptoms. This prompted the study sponsor to recommend gradual dose escalation when using a high-dose treatment regimen.

A 24-week, randomized, double-blind, placebo-controlled trial with a 24-week open-label extension involving 36 individuals with mild-to-moderate Alzheimer’s disease was conducted with curcumin C3 Complex^®^ (Ringman et al. 2012). The treatment was 2 g/day or 4 g/day administered in two divided doses with fatty meals. There were 6 withdrawals (5 from treatment groups and 1 from placebo group) due to worsening memory. There were no serious AEs reported; specific AEs included black stools, diarrhea at 2 g/day and 4 g/day. Minor clinical changes recorded included a decrease in hematocrit and increase in plasma glucose, both of which were still within normal ranges.

Lukefahr et al. (2018) reviewed clinical trials using turmeric as a dietary supplement. A review of 20 clinical trials involving 526 users of turmeric supplements showed a 5% incidence of abnormal liver function (e.g., changes in transaminases, lactate dehydrogenase, ALP, and/or bilirubin). All reported cases occurred in studies that involved >1 month of treatments. No clear relationship was found between dosage and degree of liver injury, which differs from intrinsic drug-induced liver injury (DILI). The authors concluded that the low incidence and lack of linear dose response of liver injury suggests that turmeric-associated hepatotoxicity is likely idiosyncratic.

In summary, of the clinical trials reviewed as part of the DSAEL EC admission evaluation, there was no organ toxicity or serious AEs reported. Mild and transient AEs were observed and included GI-related symptoms (nausea, diarrhea, abdominal discomfort, dyspepsia, vomiting). Other mild AEs reported were headache, dizziness, rash, dry mouth, hot flashes, pruritus, and edema. Minor hematological AEs were recorded, and these were rare and clinically insignificant (e.g., minor changes in hematocrit and plasma glucose within normal ranges). Liver and kidney function remained unaffected across trials assessing these parameters. Curcumin and curcuminoid supplementation are consistently supported as safe in the studies reviewed; however, limitations of the data on which this conclusion is based were identified, including high heterogeneity across studies including variable formulations, dosages, and durations tested. Many of the studies included in systematic reviews and meta-analyses were of low methodological quality; there were incomplete or inconsistent biochemical safety assessments. AEs were often self-reported or clinically observed and lacked standardized grading, or AE data were not collected or reported; there was limited long-term follow-up; meta-analyses did not consistently pool data relating to liver enzymes.

#### Review of adverse events case reports from adverse event report databases

Clinical trials can provide information about acute, common AEs occurring during studies, but have recognized limitations with respect to their ability to identify AEs that occur less frequently and/or that have a long latency. Spontaneous reporting systems can provide early warnings of safety concerns occurring during use of marketed products, including dietary supplements. Many countries operate such systems, usually through their medicines’ regulatory agency, and collect spontaneous reports of AEs or suspected adverse drug reactions submitted by health professionals and the general public. These data are analyzed to detect ‘signals’ of potential safety concerns. In recent years, several countries have provided some level of public access to brief anonymized information from the AE reports they have received and/or to aggregated data. The presence of reports of AEs should not be interpreted as meaning that the product(s) or substance(s) named in the report caused the AE: establishing a causal relationship is a complex process, and there may be alternative explanations for the observed adverse effects. Other limitations in relation to spontaneous reports of AEs include poor quality and missing information on aspects such as comorbidities; dechallenge and rechallenge outcomes, including effects of stopping and, where appropriate, restarting the implicated product(s); and comprehensive information on the product ingredients – particularly for dietary supplements – the health reason for its use, and treatment periods. Also, while post-marketing surveillance for dietary supplements relies almost exclusively on spontaneous reports for identifying signals of safety concerns, under-identification and under-reporting of AEs following use of dietary supplements are well-recognized issues. It is also important to understand that some systems invite reports of ‘adverse events’, and others request reports of ‘suspected adverse drug reactions’, some request reports of ‘serious reactions or illness’ from consumers and health professionals, whereas others encourage reporting of serious and non-serious adverse reactions. Thus, the data are variable across systems.

Searches using the terms ‘turmeric’, ‘curcumin’, and/or ‘*Curcuma longa*’, as appropriate (since different systems have different approaches to coding products/substances and for searching the database), were conducted in the public-facing online databases of AEs of several national reporting systems on 29 September 2025. Multiple AEs associated with turmeric or curcumin consumption were reported across several databases. The most frequently reported AEs were GI symptoms (e.g., diarrhea, nausea, abdominal pain), general and hypersensitivity reactions (e.g., rash, pruritus, anaphylaxis), nervous system complaints (e.g., headache, dizziness); a smaller number of reports were for hepatobiliary disorders, including jaundice, elevated liver enzymes, drug‑induced liver injury, autoimmune hepatitis, and acute hepatic failure.

From January 2004 to March 2025, the FDA Center for Food Safety and Applied Nutrition (CFSAN) Adverse Event Reporting System (CAERS) data files (now known as the Human Foods Complaint System) held a total of 214 cases reported in association with turmeric- or curcumin-containing dietary supplements and herbal products. GI symptoms (e.g., diarrhea, nausea, and vomiting) were among the most frequently reported AEs, along with hypersensitivity reactions, including rash, pruritus, and anaphylaxis. Cardiovascular effects, such as blood pressure fluctuations and heart rate irregularities, along with neurological complaints, such as dizziness and headache, were also reported (FDA 2025).

Regarding liver-related AEs, 17 cases of liver problems were identified involving terms such as ‘drug-induced liver injury’, ‘hepatitis’, ‘auto-immune hepatitis’, ‘hepatic enzyme elevations’, ‘jaundice’, and ‘cirrhosis’. These AEs were reported for several different products. Several cases required hospitalization and some cases were classified as life-threatening or disabling. Other symptoms reported in liver-related AEs included fatigue, malaise, abdominal pain, jaundice, and elevated liver enzymes. The majority of cases involved participants aged >55 years, although a few cases involved younger adults; 15 of the 17 liver-related cases occurred in females. One fatal case was reported, involving hepatic cirrhosis, septic shock, and esophageal hemorrhage (FDA 2025).

A search of the Canada Vigilance Adverse Reaction Online Database for the period 01 January 1965, to 31 May 2025, identified 139 adverse reaction reports for the active ingredient ‘*Curcuma longa*’, 53 reports for active ingredient ‘turmeric’, and 49 reports for active ingredient ‘curcumin’ and ‘curcuminoids’. Reported AEs included nervous system disorders, hypersensitivity reactions (e.g., anaphylactic shock, urticaria, pruritus), hepatotoxicity (e.g., jaundice, hepatic enzyme elevations), GI symptoms, cardiovascular events (e.g., palpitations, cardiac failure), and psychiatric symptoms (e.g., hallucinations, nervousness) (Health Canada Vigilance adverse reaction online database (Health Canada Vigilance Database)) 2025).

Health Canada published a summary safety review to assess the potential risk of hepatotoxicity (liver injury) associated with the oral use of turmeric- or curcuminoid-containing natural health products. The review was initiated following rare but serious international reports of liver injury. In Canada, 12 cases were identified, although all had confounding factors, including use of turmeric as part of multi-ingredient dietary-supplement formulations, or insufficient clinical data, making causality unclear. However, a link to turmeric or curcuminoids could not be ruled out. Over 60 cases (international) were also reviewed, including 3 deaths, 2 of which were attributed to turmeric-related hepatotoxicity. Based on these findings and international data, Health Canada is updating product monographs and labeling requirements for oral formulations of turmeric- and curcuminoid-containing natural health products to include warnings about liver injury risks, symptoms (e.g., jaundice, dark urine, nausea), and adding precautions for individuals with liver disorders and those taking medications (Health Canada 2025).

For Australia, a search using the term ‘curcumin’ in the Database of Adverse Event Notifications (DAEN), maintained by the Therapeutic Goods Administration (TGA), revealed 100 AE reports for the period of 01 January 1971 to 15 September 2025 and included AEs related to turmeric. GI system-related suspected AEs were the most frequently reported: diarrhea was the most frequently reported adverse reaction, followed by abdominal pain (upper), nausea, vomiting, and malaise. These symptoms were reported for products containing curcumin in combination with *Piper nigrum* (black pepper), which is claimed to enhance curcumin bioavailability. Hypersensitivity reactions were also prominent, including rash, pruritus, and even anaphylactic responses in some cases (TGA 2025).

A subset of the AE reports concerned liver-related complications associated with curcumin-containing products. Specifically, jaundice was reported in 4 cases, elevated liver function tests in 3, hepatomegaly in 1, and both acute hepatic failure and cholestatic hepatitis were each reported once. The case of acute hepatic failure involved a male patient and resulted in a fatal outcome. Additionally, the term ‘liver function test abnormal’ appeared in at least 3 separate entries. These reactions were reported for curcumin products often used alongside other supplements or medications. In total, at least 10 cases of liver-related AEs were identified for curcumin-containing products. (TGA 2025).

A separate search in the DAEN for ‘*Curcuma longa*’ identified 332 reports, including 274 cases linked to a single suspected medicine and 1 reported death [Note: cases involving products labeled as containing both ‘curcumin’ and ‘*Curcuma longa*’ will appear in both sets of search results]. Most AE reports involved GI symptoms (e.g., nausea, diarrhea, upper abdominal pain, vomiting, and malaise, along with fatigue, headache, sleep disturbances, and hypersensitivity reactions. Hepatobiliary disorders accounted for 14 reports (12 female, 2 male), while investigations (elevated hepatic enzymes) accounted for 12 cases (all female). Six cases overlapped between these categories, resulting in a total of 20 hepatobiliary-related AE reports (18 in females and 2 in males) (TGA 2025).

In the New Zealand Medsafe Suspected Medicines Adverse Reactions Search database (NZ Medsafe 2025), 2 cases were identified using the search term ‘curcumin’. The reported ADRs included nephrolithiasis, hepatomegaly, acne, gynecomastia, and deep vein thrombosis. A total of 30 ADR reports were identified for another search for term ‘turmeric’. In total, 5 cases described liver-related AEs following use of single-ingredient turmeric supplements and multi-ingredient formulations, usually alongside other medications.

A search using the term ‘Curcuma longa’ in the VigiAccess WHO ADR database revealed 530 ADRs (VigiAccess). VigiAccess is a web-based tool that allows searching of VigiBase, the WHO’s global database of AE reports for medicines and vaccines. VigiBase does not directly accept reports from reporters; rather, reports are submitted to VigiBase by national pharmacovigilance centers or national drug regulatory authorities in countries contributing to the WHO’s Program for International Drug Monitoring (PIDM). Over half of the 530 reports were submitted to VigiBase by national pharmacovigilance centers or national drug regulatory authorities in countries in the WHO Asia region that contribute to the WHO’s PIDM; the remainder came from WHO PIDM participating countries in North and South America, Europe, Africa, and Oceania. From reviewing the numbers of reports for each of the countries discussed earlier in this section, it appears that VigiAccess does not hold all the reports that can be identified on the national pharmacovigilance center websites for those countries.

The most frequently reported ADR categories were general disorders and administration site conditions (218 cases), GI disorders (190 cases), skin and subcutaneous tissue disorders (112 cases), and nervous system disorders (99 cases). There were 34 reports that included AE terms relating to hepatobiliary disorders, with serious liver-related events including jaundice (9 cases), hepatic cytolysis (5 cases), autoimmune hepatitis (4 cases), drug-induced liver injury (3 cases), hepatotoxicity (3 cases), and acute hepatic failure (2 cases). Additional reports included hepatic cirrhosis, fibrosis, necrosis, steatosis, and portal hypertension (VigiAccess 2025). VigiAccess does not allow searching by participating countries and so it is not possible to determine whether the reports in VigiBase are those submitted by national pharmacovigilance centers of countries discussed earlier in this section, or whether some, or all, of them were submitted by other countries, such as those in the WHO Asia or Africa regions.

AE data extracted from databases discussed in this manuscript are derived from spontaneous reports and reflect information submitted by reporters, without verification of accuracy or completeness. These datasets frequently contain incomplete, unconfirmed, or duplicate information, cannot be used to determine incidence, prevalence, or comparative risk, and the existence of reports does not necessarily establish causality between a product and an adverse event. Reported events may be influenced by factors such as underlying disease, concomitant product use, reporter interpretation, or coincidental timing. Therefore, these databases are intended solely for signal detection and hypothesis generation, and should not be used in isolation to assess product safety or draw causal conclusions.

#### Case reports in peer-reviewed literature

More than 30 individual published case reports and several case series have suggested an association between turmeric- and/or curcuminoid- formulations often combined with piperine to DILI. For example, Menniti-Ippolito et al. (2020) reported 28 cases of acute hepatitis associated with turmeric supplements. Details of case reports are summarized in Table 3. Most cases in the published literature involved women spanning young to older adulthood (Menniti-Ippolito et al. 2020; Halegoua‑DeMarzio et al. 2023; Papke et al. 2025). It is not clear whether all of the published case reports were also submitted to the respective national pharmacovigilance centers as spontaneous reports.

**Table 3.** Case reports of hepatotoxicity associated with oral consumption of turmeric or curcumin.

Year (Event occurred)	Age (years)	Gender	Preparation	Dosage	Length of use	Piperine use	Concomitant drugs and supplements	Diagnosis	CIOMS/RUCAM score	Outcome	References
2015	78	F	Turmeric supplement	NR	NR	NR	Etoricoxib	DILI/HILI	NR	Recovered	Fernández‑Aceñero et al. (2019)
2017 (reported; event year not specified)	45	F	Curcumin supplement; specific preparation not described	NR	NR	NR	Lisinopril, levothyroxine, atorvastatin, duloxetine, curcumin, dehydroepiandrosterone (DHEA), multivitamin; also on hormonal treatments for *in vitro* fertilization attempt	Drug-induced granulomatous hepatitis; biopsy with multiple non-necrotizing granulomas	NR	Recovered	Lim and Sundaram (2017)
2018 (reported; event year not specified)	71	F	Turmeric dietary supplement	NR	≈12 months	NR	Amlodipine; metoprolol; atenolol; benazepril; levothyroxine; meloxicam; estradiol; loratadine; diphenhydramine; aspirin; calcium; vitamin D; multivitamin; fish oil; proprietary alpha-galactosidase and invertase; lysine; alfalfa powder; glucosamine with chondroitin; proprietary high-fiber supplement; proprietary vitamins, minerals and grape seed extract; prior red yeast rice discontinued before event	Drug-induced autoimmune hepatitis	7, ‘probable’ (RUCAM)	Recovered	Lukefahr et al. (2018)
2017	52	F	Ancient Wisdom Modern Medicine High Potency Turmeric	375 mg/day (curcuminoids)	∼1 month	4 mg/day black pepper	Flaxseed oil; occasional diclofenac; cholecalciferol 50 mcg daily; ascorbic acid 500 mg daily; levonorgestrel 52 mg IUD; at re-challenge turmeric was used as sole therapy	DILI/HILI	9 (highly probable)	Recovered	Luber et al. (2019)
2017 (reported; event year not specified)	55	M	Turmeric supplement	NR	5 months	NR	Telmisartan; Atenolol; Lercanidipine; background idiopathic thrombocytopenic purpura, gout, osteoarthritis	DILI/HILI	6 (probable)	Recovered	Luber et al. (2019)
2017 (reported; event year not specified)	61	F	Turmeric supplement	NR	6 months	NR	Naproxen; ergocalciferol	DILI/HILI	8 (probable)	Recovered	Suhail et al. (2020)
2019 (reported; event year not specified)	78	F	Turmeric herbal supplement	500 mg/day	∼1 month	NR	Aspirin, citalopram, losartan, metformin, oxybutynin, (simvastatin stopped one month earlier)	Hepatocellular DILI with painless jaundice	6 (probable)	Recovered	Imam et al. (2019)
2020 (reported; event year not specified)	55	F	Qunol Liquid Turmeric	15 mL/day (mg not specified)	3 months	Yes black pepper extract/piperine present)	Levothyroxine 50 mg daily; famotidine 20 mg daily; aluminum hydroxide–magnesium hydroxide–simethicone oral suspension	Drug‑induced autoimmune hepatitis (DI‑AIH) AIH score 18 (definite)	9 (highly probable)	Recovered	Lee et al. (2020)
2020 (reported; event year not specified)	62	F	Turmeric tablets	NR	≈10 months	NR	Hypoglycemic medication and hormonal therapy	DILI/HILI	NR	Recovered	Chand et al. (2020)
2020 (reported; event year not specified)	51	F	OTC turmeric dietary supplement	One capsule daily (400 mg turmeric powder + 50 mg turmeric extract + 50 mg organic ginger powder)	2 months	No	Sertraline 25 mg daily; OTC multivitamin; primrose oil; omega‑3; infrequent acetaminophen; short course methylprednisolone and cetirizine	DILI/HILI	Probable (RUCAM), numeric score NR	Recovered	Abdallah et al. (2020)
2021 (reported; event year not specified)	57	F	OTC turmeric dietary supplement	2000 mg/day	3 months	Yes	Omeprazole, metoprolol, atorvastatin, aspirin, fish oil, tamsulosin, vitamin D	DILI/HILI	7 (probable)	Recovered	Sohal et al. (2021)
2021 (reported; event year not specified)	53	F	Turmeric tablet	NR	NR	NR	Long‑term apple cider vinegar use	DILI/HILI	6 (probable)	Recovered	Sohal et al. (2021)
2021 (reported; event year not specified)	53	F	Multi-ingredient herbal regimen	30 mg/day turmeric per label serving	3-4 weeks	No	Multi‑ingredient herbal regimen: NOW Foods Liver Detoxifier and Regenerator (includes turmeric root 30 mg per serving among 16 herbal ingredients) plus Amen Solutions Restful Sleep (valerian‑based)	HILI ((cholestatic pattern; authors discuss valerian and scute as more likely contributors; turmeric present at low dose)	6 (probable)	Recovered	Koenig et al. (2021)
2022 (reported; event year not specified)	63	F	OTC turmeric dietary supplement	NR	6 weeks	NR	Patient denied other medications	DILI/HILI	9 (highly probable)	Recovered	Ashraf et al. (2022)
2022 (reported; event year not specified)	49	F	Turmeric supplement	1000 mg/day	3 months	Yes	NR (no chronic meds listed)	DILI/HILI (patient restarted turmeric a few weeks after discharge leading to positive rechallenge and severe recurrence)	NR	Recovered	Liu and Chang (2022)
2022 (reported; event year not specified)	65	F	Turmeric supplement	500 mg/day	Initial episode: NR; Re‑challenge: 3 weeks before recurrence	NR	Aspirin 81 mg, rosuvastatin 5 mg, niacinamide 500 mg, and various vitamins/supplements; all meds/supplements stopped at first presentation; later turmeric alone was restarted before recurrence	DILI/HILI (positive re-challenge)	NR	Recovered	Lopez et al. (2022)
2022 (reported; event year not specified)	49	F	Label listed turmeric containing curcuminoids and piperine	1,000 mg (950 mg curcuminoids)/day	∼1.5 months	Black pepper 10 mg/day (piperine 9 mg/day)	*Lactobacillus rhamnosus*; vitamin C; latanoprost and timolol ophthalmic solutions; levonorgestrel IUD; single brief use of an aspirin‑caffeine‑acetaminophen combination tablet one week prior	DILI/HILI	7 (probable)	Recovered	Sunagawa et al. (2022)
2023 (reported; event year not specified)	62	F	Turmeric Tea	NR	3 weeks	NR	Hydrochlorothiazide	DILI/HILI	9 (highly probable)	Recovered	Smith et al. (2023)
2023 (reported; event year not specified)	55	F	Turmeric supplement	1500 mg/day	∼1 month	Yes	Moderate alcohol use (≈2 glasses wine/day; binge 5 drinks 5 days prior); no regular medicines reported	DILI/HILI	9 (highly probable)	Recovered	Ajitkumar et al. (2023)
2023 (reported; event year not specified)	36	F	Qunol liquid turmeric	2,000 mg curcumin extract/day per label	6 months	Yes	None reported (no prescription/OTC medicines, minimal alcohol)	DILI/HILI	NR	Recovered	Smati et al. (2023)
2023	35	F	OTC Curcumin tablet	Curcumin ∼120 mg/day	2 months	Black pepper ∼20 mg/day	Occasional alcohol; no other drugs or vaccinations reported	DILI/HILI	8 (probable)	Recovered	Garaizabal Azkue et al. (2024)
2023 (reported; event year not specified)	28	M	Daily TURMERIC+ (Scientific Nutrition) supplement; ingredients include turmeric, ginger, Bioperine^®^ black pepper	NR	∼5 months	Yes	Recent cocaine and alcohol 5 days pre‑admission	Drug-induced autoimmune hepatitis (DI-AIH)	6 (probable)	Recovered	Arzallus et al. (2023)
2024 (reported; event year not specified)	53	F	Kirkland Signature Turmeric daily	Turmeric 1,000 mg/day	5 months	Black pepper 10 mg/day	Sertraline 200 mg daily (long‑term); 3–4 beers on weekend	DILI/HILI	Naranjo = 7 (probable)	Recovered	Abboud et al. (2024)
2024 (reported; event year not specified)	59	F	Turmeric supplement	NR	4 months	Yes	No chronic medicines noted	DILI/HILI	NR	Recovered	Jalalian et al. (2024) Conference abstract describing the same case later reported in detail by Spitofsky et al. (2025)
2024 (reported; event year not specified)	NR (pregnant adult)	F	Turmeric powder	3–4 times/day, 5–10 g per intake	Short term (exact duration NR)	NR	Ursodeoxycholic acid (250 mg QID initially for pruritus/cholestasis) during pregnancy	Probable DILI/HILI	NR	Recovered Postpartum liver function tests normal; delivered a healthy infant at 37 weeks	Haloub et al. (2024)
2024 (reported; event year not specified)	70	F	OTC turmeric capsules: 1500 mg turmeric root extract (95% curcuminoids) + 100 mg organic ginger + 10 mg black pepper per capsule	3000 mg/day turmeric root extract	∼2 months	Black pepper 20 mg/day	Semaglutide 2 mg weekly started ∼2 months prior (for weight loss); multiple chronic medicines (e.g., sertraline, spironolactone, nortriptyline, temazepam)	DILI/HILI	8 (probable)	Recovered	Zhang et al. (2024)
2025 (reported; event year not specified)	59	F	OTC turmeric + black pepper supplement	Turmeric ∼2,320 mg/day	∼4 months	Black pepper 10 mg/day	None (azithromycin 3 months earlier for resolved diarrheal illness)	DILI/HILI	7 (probable)	Recovered	Spitofsky et al.(2025)
2025 (reported; event year not specified)	40	F	Self‑made ‘wellness shots’: ground turmeric + black pepper + fresh orange juice + apple cider vinegar + water; amounts not quantified	NR	5 months	Yes	Oral contraceptive pill long‑term	DILI/HILI	6 (probable)	Recovered	Shrestha et al. (2025)
Enrolled in DILIN between 2004–2022; event year not specified)	57	F	Nature’s Way Turmeric, OTC capsule	500 mg/day	2–3 weeks	No	Rizatriptan, salbutamol, vitamin C, vitamin D, multivitamin	Acute hepatitis with jaundice, mixed pattern	NR	Recovered	Halegoua‑DeMarzio et al. (2023); NIH/NIDDK (2025)
Enrolled in DILIN between 2004–2022; event year not specified)	35	M	Curcumin C3 (Nature’s Lab) with BioPerine^®^	1000 mg/day curcumin	∼2 months	5 mg piperine/day	Ibuprofen, ‘Move Free Joint Support’ (glucosamine, chondroitin, Boswellia), collagen powder, fish oil, vitamins B and C, multivitamin	Hepatocellular DILI with jaundice	NR	Recovered	Halegoua‑DeMarzio et al. (2023); NIH/NIDDK (2025)
Enrolled in DILIN between 2004–2022; event year not specified)	62	F	Rite Aid Turmeric root extract	500 mg/day	14 months	No	Estrogens, pseudoephedrine, diphenhydramine, salbutamol, tramadol, magnesium, fish oil, ginger, glucosamine, vitamins C and D, multivitamin	Severe hepatocellular DILI progressing to acute liver failure	Probable (qualitative)	Death (multiorgan failure while awaiting transplant)	Halegoua‑DeMarzio et al. (2023); NIH/NIDDK (2025)
2024 (reported; event year not specified)	49	F	Provitalize menopause supplement containing turmeric root extract (95% curcuminoids) 350 mg, black pepper fruit extract (BioPerine^®^) 3 mg per capsule along with other ingredients	Turmeric extract ∼350 mg/day	∼30 days	Black pepper 3 mg/day	History of asthma on no chronic medications. Other ingredients in Provitalize menopause supplement: Probiotic blend (*B. breve, L. gasseri*, *B. lactis*), moringa leaf 350 mg, curry leaf 150 mg, lecithin 50 mg	DILI/HILI (Acute liver failure)	NR	Death before transplant	Patel et al. (2024)
2024	66	F	Ground turmeric powder from local herbal store	Half‑teaspoon daily; mg not quantified	∼6 months initial use, stopped and resumed later leading up to admission	NR	Long-term cardiovascular medications (metoprolol, amlodipine, hydralazine, lisinopril, atenolol, chlorthalidone, aspirin, clopidogrel, atorvastatin) unchanged for ≥1 year prior; all stopped on admission. Medical history included peripheral arterial disease, stage two chronic kidney disease, and a newly diagnosed breast cancer awaiting chemotherapy initiation	DILI/HILI	5 (possible)	Death (complicated by acute liver failure and hepatorenal syndrome)	Alghzawi et al. (2024)

*NR: Not Reported.

Causality assessments using the Roussel Uclaf Causality Assessment Method (RUCAM) yielded scores between 5 and 9, indicating ‘possible’ to ‘highly probable’ associations. Dosages varied widely, mostly within the range of 30 mg to 3000 mg per day of turmeric or curcuminoid preparations in supplements (Table 3) with durations of use ranging from two weeks to over one year. Clinical presentations commonly included jaundice, fatigue, nausea, pruritus, and elevated liver enzymes, with ALT and AST often exceeding 1000 U/L. Histopathological findings ranged from acute hepatitis and interface hepatitis to autoimmune-like features and cholestasis. Several cases confirmed turmeric-induced liver injury through positive rechallenge, and some met diagnostic criteria for autoimmune hepatitis (AIH), suggesting drug-induced AIH (DI-AIH). Genetic predisposition, particularly the HLA-B*35:01 allele, was observed in several cases and may contribute to immune-mediated hepatotoxicity (NIH/NIDDK, 2025). Piperine, often included to claim enhanced curcumin absorption, was implicated in many cases (Sohal et al. 2021; Liu and Chang 2022; Ajitkumar et al. 2023).

Limitations of published case reports/case series included inconsistent product analysis, confounding factors, such as concurrent medications and alcohol use, and incomplete clinical data. Mechanistically, turmeric-induced liver injury appears idiosyncratic and immune-mediated, with hypotheses involving enhanced bioavailability, mitochondrial dysfunction, and oxidative stress.

Using the National Health and Nutrition Examination Survey’s (NHANES) 2017–March 2020 weighted data and 2020 U.S. Census totals, Likhitsup et al. (2024) estimated that approximately 11.4 million U.S. adults reported turmeric/curcumin use in the past 30 days. Users were, on average, 52.2 years old, 51.2% were female, and most reported taking turmeric/curcumin for general health, immunity, or arthritis (26.8%). Additionally, 87.6% used these substances without clinician recommendation. Liver‑related outcomes were similar between turmeric/curcumin users and non‑users, with self‑reported liver disorders occurring in 2.7% of users versus 3.5% of non‑users, and mean ALT (22.7 vs. 23.4 U/L) and ALP (71.8 vs. 77.4 U/L) concentrations remaining within normal ranges. The analysis revealed no overt liver toxicity in the general population, though caution remains appropriate due to unregulated supplement quality and the potential for rare idiosyncratic injury (Likhitsup et al. 2024).

Overall, occasional AEs with turmeric- and/or curcuminoid supplements may therefore reflect rare idiosyncratic or genetic factors rather than dose-dependent reactions; contamination has been considered but is judged unlikely in UK surveys (UK COT 2024; TGA 2023a; Stati et al. 2021). The National Institutes of Health (NIH) LiverTox database now recognizes turmeric as a well-documented cause of herbal-related liver injury in the U.S. and assigns it a likelihood score of ‘A’, indicating a recently established and well-supported cause of clinically apparent liver injury (NIH/NIDDK 2025).

### Regulatory action and labeling requirements

Turmeric, when consumed as part of the diet, is generally considered safe. However, concentrated extracts of turmeric or curcuminoids used as supplements may require a cautionary statement. A review of turmeric or curcumin containing product labels using online dietary supplement product databases or registries (e.g., Dietary Supplement Label Database, *Supplement OWL*^®^) reveals cautionary language including statements directed at individuals with liver-related issues as well as calling out potential interaction with drugs ([DSLD] Dietary Supplement Label Database 2026; Supplement OWL (Online Wellness Library) 2026). Common warnings or cautionary statements include language such as ‘not recommended for individuals with gallbladder issues, bile duct obstruction, or gastrointestinal disorders’. Product labels also caution against exceeding the recommended dose or mixing with other drugs or supplements without medical advice over concern for potential for drug interaction.

Product labels also mention possible side effects such as nausea, vomiting, abdominal pain, jaundice, dark urine, and loss of appetite, recommending discontinuation and medical consultation if these occur. Overall, these cautionary statements reflect the need for careful use and medical guidance, especially for vulnerable groups or when combined with medications.

Several regulatory agencies and authoritative scientific bodies internationally have issued safety advisories and/or have made cautionary labeling recommendations or requirements related to turmeric- and/or curcuminoid-containing supplements as a result of concerns associated with a potential link between the use of these ingredients and hepatotoxicity (Table 4). In some cases, country-specific monographs have been updated to include such cautionary statements.

**Table 4.** Regulatory agencies and authoritative scientific bodies internationally that recommended or required liver-related cautionary labeling for turmeric- and curcuminoid-containing products.

Source	Cautionary statement
WHO	*Contraindicated in biliary obstruction, gallstones, and hypersensitivity*
ANSES (France) and Italian Ministry	*Caution against use in liver conditions; prohibit hepatic/joint health claims*
Australian TGA	*In very rare cases, Curcuma species/Curcumin may harm the liver. Stop use and see a doctor if you have yellowing skin/eyes or unusual: fatigue, nausea, appetite loss, abdominal pain, dark urine, or itching.*
Health Canada	*Ask a health care practitioner/health care provider/health care professional/doctor/physician before use if you have a liver or biliary disorder. Ask a health care practitioner/health care provider/health care professional/doctor/physician before use if you are taking medications. Stop use and ask a health care practitioner/health care provider/health care professional/doctor/physician if you experience any new symptoms including yellowing of the eyes or skin, dark urine, nausea, vomiting, stomach pain.*

The European Medicines Agency (EMA) Committee on Herbal Medicinal Products (HMPC) issued an EU herbal monograph in 2018 recognizing *C. longa* L. rhizome preparations as traditional herbal medicinal products for adults with mild digestive disturbances, with defined oral preparations (powdered or comminuted rhizome, tinctures, and specified ethanol dry extracts) (EMA 2018a; EMA 2018b). The same EMA/HMPC monograph advises that use is not recommended in bile duct obstruction, cholangitis, liver disease, gallstones, and other biliary diseases, and recommends medical consultation if symptoms persist beyond two weeks (EMA 2018b). In Italy, the Ministry of Health (2019) introduced mandatory label warnings for food supplements containing *Curcuma* preparations (Decree of 26 July 2019; compliance by 31 December 2019) stating that use is not recommended in the event of altered liver function or biliary or gallstone disorders and advising medical consultation when medicines are used concomitantly (Italian Ministry of Health (Ministero della Salute)) 2019a, 2019b). Italy subsequently strengthened these measures in 2022, requiring an ‘important warning’ that reiterates liver and biliary cautions and adds advice not to use during pregnancy and lactation and not to use for prolonged periods without medical advice, and it also reports removal of previously allowed ‘physiological effect’ claims, with label compliance by 31 December 2022 (Italian Ministry of Health (Ministero della Salute)) 2022). Germany’s Federal Institute for Risk Assessment (Bundesinstitut für Risikobewertung, BfR. German Federal Institute for Risk Assessment (BfR)) (2021) warned that curcumin intake from supplements could exceed the ADI of 3 mg/kg body weight/day derived for curcumin as a food additive (E100) and emphasized that prolonged exceedance is undesirable (BfR. German Federal Institute for Risk Assessment (BfR) 2021). The BfR. German Federal Institute for Risk Assessment (BfR) (2021) further noted that piperine and other bioavailability-enhancing technologies can increase exposure, so risk assessment should be product-specific, including consideration of reported liver-related effects particularly for enhanced-bioavailability preparations. In France, the French Agency for Food, Environmental and Occupational Health and Safety (Agence nationale de sécurité sanitaire de l’alimentation, de l’environnement et du travail, ANSES) (2022) reported more than 100 nutrivigilance AE reports (including hepatitis) potentially linked to turmeric/curcumin supplements and recommended keeping supplement-derived curcumin intake below 153 mg/day for a 60 kg adult, noting additional concern for formulations designed to increase bioavailability (e.g., piperine combinations and advanced delivery systems) (ANSES 2022a; 2022b). In Australia, the TGA (2023) issued a safety advisory stating that turmeric-/curcumin-containing medicines/supplements may cause rare, but sometimes severe, liver injury, and that the risk may be higher for high-dose and/or enhanced absorption/bioavailability products (TGA 2023b). The UK Committee on the Toxicity of Chemicals in Food, Consumer Products and the Environment (COT) concluded in its statement COT/2024/07 (updated 9 December 2024) that, despite limited data, there is reasonable evidence linking turmeric (including supplement use) to liver toxicity, because reported adverse effects occurred on exposure and improved after product withdrawal, consistent with an idiosyncratic reaction. COT noted that dietary intake from food additive or culinary use is generally within the ADI (0–3 mg/kg bw/day), while supplement use can cause occasional exceedances; minor exceedances based on label directions were judged unlikely to pose significant risk for most people, but substantial exceedances represent a potential health risk, especially with concomitant medicines or altered hepato-biliary function. COT further emphasized that, rarely, individuals may experience liver injury with exposures even below the ADI (potentially influenced by genetic susceptibility) and recommended further assessment of novel formulations (e.g., micellar/nano/micro systems) that may alter pharmacokinetics and bioavailability (UK COT 2024). Finally, Health Canada’s Natural and Non-prescription Health Products Directorate (NNHPD) updated its monograph for concentrated turmeric extracts and isolates (curcuminoids/curcumin), specifying that extracts standardized to ≥75% curcuminoids should not exceed 1500 mg curcuminoids/day (500 mg per single dose), and noting that enhanced-absorption dosage forms/formulations are not covered by NNHPD monographs and should be submitted as Class III applications with supporting evidence; the monograph also includes liver- and biliary-related cautions and stop-use advice if symptoms suggestive of liver injury occur (Health Canada 2025; Health Canada NNHPD 2025).

Overall, this review synthesizes evidence across clinical trials, non-clinical toxicology, pharmacokinetics/ADME, case reports, spontaneous AE databases, product-label sources, and regulatory actions to evaluate hepatic safety signals for turmeric (*C. longa*) and curcuminoids in contemporary supplement contexts. Turmeric and curcuminoid products are marketed in diverse dosage forms and in claimed bioavailability-enhanced formulations, making rigorous physical, chemical, and microbiological quality control essential because commercial products can vary in composition and contain impurities, which complicates exposure characterization and interpretation of safety outcomes. The review includes largely reassuring clinical trial safety syntheses, and pharmacovigilance and case-series evidence indicating a predominantly hepatocellular injury phenotype that typically improves after product discontinuation but rarely culminates in acute liver failure, with proposed mechanisms consistent with idiosyncratic immune-mediated injury and possible genetic susceptibility. Regulatory and labeling considerations were also integrated, culminating in a proposed cautionary statement to strengthen risk communication, particularly for individuals with liver-related susceptibility and to facilitate prompt recognition of symptoms suggestive of liver injury.

## Limitations

The USP admission evaluation focuses on potential safety concerns and does not evaluate evidence relating to efficacy; accordingly, the USP admission evaluation process addresses solely the harms component of the overall benefit-harm balance. Evidence from umbrella reviews, systematic reviews, and meta-analyses of clinical trials that reported safety information was considered, and a summary of DSAEL EC findings is presented; however, individual clinical trials were not systematically screened for safety outcomes and no formal risk-of-bias assessments were performed. Comprehensive capture of preclinical or clinical ADME investigations on turmeric and curcuminoid formulations was not feasible given the rapidly expanding array of products and delivery systems. Access to complete case-level information for spontaneous reports and published case reports was unavailable, precluding independent verification of exposure, product identity, dose, co-exposures, and timing, as well as standardized causality assessment. In addition, exposure information frequently relies on self-report and product labels that may not reflect actual constituents or bioavailability, and formulations, doses, and co-ingredients vary widely, limiting generalizability. However, the USP admission evaluation is a continuous, iterative process and will be revised as newer and higher-quality evidence emerges.

## Concluding remarks

Turmeric is widely used as a spice with average dietary intake in some regions of the world of up to 2.5 g/day (equivalent to approximately 100 mg curcumin daily). To date, there remain no safety concerns associated with the use of turmeric as a food ingredient. Turmeric has a long history of traditional use across Asia, including in Ayurvedic, Unani, Siddha, and East Asian medicine systems, for treating digestive, hepatic, inflammatory, and skin conditions. It is officially recognized in global pharmacopoeias, and the WHO monograph and The Complete German Commission E monographs support its use for dyspepsia and inflammation. Contemporary marketed turmeric and curcumin supplements often contain standardized extracts with enhanced bioavailability, and commercial products make a range of health claims related to joint, liver, heart, immune, and cognitive health, though many lack clinical substantiation.

Despite a long history of safe use and consumption of turmeric, recent case reports of hepatotoxicity associated with use of contemporary turmeric-/curcuminoid-containing supplement products have emerged and have resulted in increased scrutiny from drug regulatory agencies and authoritative experts. Several regulatory reviews and expert opinions have resulted in new labeling requirements for turmeric-containing products. Alongside these developments, the USP DSAEL EC conducted a comprehensive evaluation of turmeric and curcuminoids in order to advise on any required updates to existing USP monographs.

Hepatotoxicity has been observed with turmeric in repeated dose rodent toxicology studies, but only at dose levels far in excess of estimated human uses. No organ toxicity or serious AEs have been reported in numerous clinical trials that have been conducted with turmeric and/or curcuminoids. Liver function was assessed in several of these clinical trials without any reported effects on liver parameters. However, limitations were identified in these clinical trials, including high heterogeneity across study parameters, low methodological quality, incomplete or inconsistent assessment of biochemical parameters, and AEs that were often self-reported or lacked standardized grading.

Case reports that have linked turmeric to clinically apparent liver injury include formulations containing piperine (black pepper) or multiple other ingredients. In case reports, hepatotoxicity typically occurs after 1 to 4 months of use and presents with symptoms such as fatigue, nausea, dark urine, and jaundice. Laboratory findings typically show markedly elevated aminotransferases (ALT often greater than 1,000 U/L), mild elevation in alkaline phosphatase, and occasional autoantibody positivity. Histological features may resemble autoimmune hepatitis. Most cases resolve within 1 to 3 months after discontinuation, although rare instances of acute liver failure requiring transplantation have been reported. In one case series, the proposed mechanism may be idiosyncratic and immune-mediated, and emerging research has also linked curcumin-related liver injury to a strong genetic association with the HLA-B*35:01 allele (Halegoua‑DeMarzio et al. 2023).

As a result of these case studies many global regulatory authorities have associated a link between use of turmeric and curcuminoid supplements and risk of liver injury. Due to the rare, but serious outcomes in some cases, global authorities have recommended or required cautionary labeling on turmeric- and curcumin-containing products regarding potential hepatotoxicity. After completing an admission evaluation for turmeric and curcuminoids, the USP DSAEL EC recommended the following cautionary label be added to the current USP monographs for turmeric and curcuminoids: *Liver problems have been reported very rarely in people taking supplements containing turmeric and/or curcuminoids. Consult your health-care practitioner before using this product if you have a history of liver problems. Stop using this product if you develop symptoms such as abdominal pain, dark urine, or jaundice (yellowing of the skin or eyes), and seek medical advice*.

This substantive narrative review of published literature concerning the pharmacokinetic, preclinical, toxicological and clinical data relating to the safety of turmeric and curcuminoids, with a focus on hepatotoxicity, has relevance across several disciplines, including chemistry, toxicology, medicine, pharmacy and public health more broadly. Given the safety concern with turmeric and curcuminoid-containing products, the review is particularly pertinent for individuals working in the dietary supplements industry, medicines and health products regulation, health professionals and other stakeholders in order to ensure avoidable harm to consumers of dietary supplement products.

## Data Availability

All raw data from original research papers and on-line databases are summarized in this review article, and the data sources are cited in the Reference section.
